# State-of-the-art diagnosis of autoimmune blistering diseases

**DOI:** 10.3389/fimmu.2024.1363032

**Published:** 2024-06-06

**Authors:** Nina van Beek, Maike M. Holtsche, Ingeborg Atefi, Henning Olbrich, Marie J. Schmitz, Jasper Pruessmann, Artem Vorobyev, Enno Schmidt

**Affiliations:** ^1^ Department of Dermatology, Allergology and Venerology, University of Lübeck, Lübeck, Germany; ^2^ Lübeck Institute of Experimental Dermatology, University of Lübeck, Lübeck, Germany

**Keywords:** autoimmune bullous disease, pemphigoid, pemphigus, autoantibody, immunofluorescence, epidermolysis, dermatitis herpetiformis, linear IgA dermatosis

## Abstract

Autoimmune blistering disorders (AIBDs) are a heterogeneous group of approximately a dozen entities comprising pemphigus and pemphigoid disorders and dermatitis herpetiformis. The exact diagnosis of AIBDs is critical for both prognosis and treatment and is based on the clinical appearance combined with the detection of tissue-bound and circulating autoantibodies. While blisters and erosions on the skin and/or inspectable mucosal surfaces are typical, lesions may be highly variable with erythematous, urticarial, prurigo-like, or eczematous manifestations. While direct immunofluorescence microscopy (IFM) of a perilesional biopsy is still the diagnostic gold standard, the molecular identification of the major target antigens opened novel therapeutic avenues. At present, most AIBDs can be diagnosed by the detection of autoantigen-specific serum antibodies by enzyme-linked immunosorbent assay (ELISA) or indirect IFM when the clinical picture is known. This is achieved by easily available and highly specific and sensitive assays employing recombinant immunodominant fragments of the major target antigens, i.e., desmoglein 1 (for pemphigus foliaceus), desmoglein 3 (for pemphigus vulgaris), envoplakin (for paraneoplastic pemphigus), BP180/type XVII collagen (for bullous pemphigoid, pemphigoid gestationis, and mucous membrane pemphigoid), laminin 332 (for mucous membrane pemphigoid), laminin β4 (for anti-p200 pemphigoid), type VII collagen (for epidermolysis bullosa acquisita and mucous membrane pemphigoid), and transglutaminase 3 (for dermatitis herpetiformis). Indirect IFM on tissue substrates and in-house ELISA and immunoblot tests are required to detect autoantibodies in some AIBD patients including those with linear IgA disease. Here, a straightforward modern approach to diagnosing AIBDs is presented including diagnostic criteria according to national and international guidelines supplemented by long-term in-house expertise.

## Introduction

Autoimmune blistering diseases (AIBDs) are a heterogeneous group of mainly autoantibody-driven diseases presenting with blisters and/or erosions on the skin and/or mucous membranes (1). AIBDs can be categorized into pemphigus and pemphigoid diseases as well as dermatitis herpetiformis. Pemphigus diseases are immunopathologically characterized by intraepidermal split formation and acantholysis, i.e., keratinocytes that have lost cell–cell contact as well as autoantibodies against desmosomal proteins (2–4). In pemphigoid disorders, autoantibodies bind to structural proteins of the basement membrane zone (BMZ) of the skin and surface close epithelia inducing subepidermal/subepithelial splitting (5, 6). Dermatitis herpetiformis is also classified as subepidermal blistering AIBDs, while autoantibodies are directed against two enzymes, transglutaminase (TG) 2 and 3 (7, 8). In Western countries, bullous pemphigoid is the by far most common AIBDs with incidences ranging from 13 to 42 patients per million inhabitants per year (9–12). It affects mainly elderly patients with an increase in incidence from the age of 70 years (11, 13–15). In contrast, pemphigus diseases, mainly pemphigus vulgaris, are more common in Middle Eastern countries with incidences between 0.8 in Finland and 16.1 in Israel (9, 16, 17). In children, linear IgA disease is reported as the most frequent entity (18).

Therapy of AIBDs is based on different degrees of immunosuppression flanked by supportive therapy. While in bullous pemphigoid super-potent topical corticosteroids are preferred due to their favorable safety profile in this elderly patient population, in pemphigus, systemic corticosteroids at higher doses of 1–1.5 mg/kg body weight and the anti-CD20 antibody rituximab are recommended (19–21).

During the last decades, deciphering the molecular identity of the individual target antigens led not only to a better understanding of the pathophysiology but also to increasingly defined entities within AIBDs. These are associated with distinct clinical and/or pathological features such as needing long-term intensive immunosuppressive therapy in epidermolysis bullosa acquisita, an association with tumors in paraneoplastic pemphigus and anti-laminin 332 mucous membrane pemphigoid (MMP), or fast response to a gluten-free diet and dapsone treatment in dermatitis herpetiformis (1, 22–25). While direct immunofluorescence microscopy (IFM) is considered the gold standard for diagnosing AIBDs, for most targeted antigens, assays to detect the respective serum autoantibodies have been developed and allow exact identification of the disease entity. Here, we summarize the current state-of-the-art diagnostic approach for AIBDs.

This review is dedicated to Detlef Zillikens, professor and chair of the Department of Dermatology, University of Lübeck, Germany. He passed away in office after a short and severe disease in September 2022 (26). He established the routine autoimmune laboratory in Lübeck, inspired the introduction of new assays and methods, and enormously contributed to the field of diagnosis of AIBDs. All authors are greatly indebted to him; he has been our mentor and friend.

## Clinical picture

**Figure 1:**
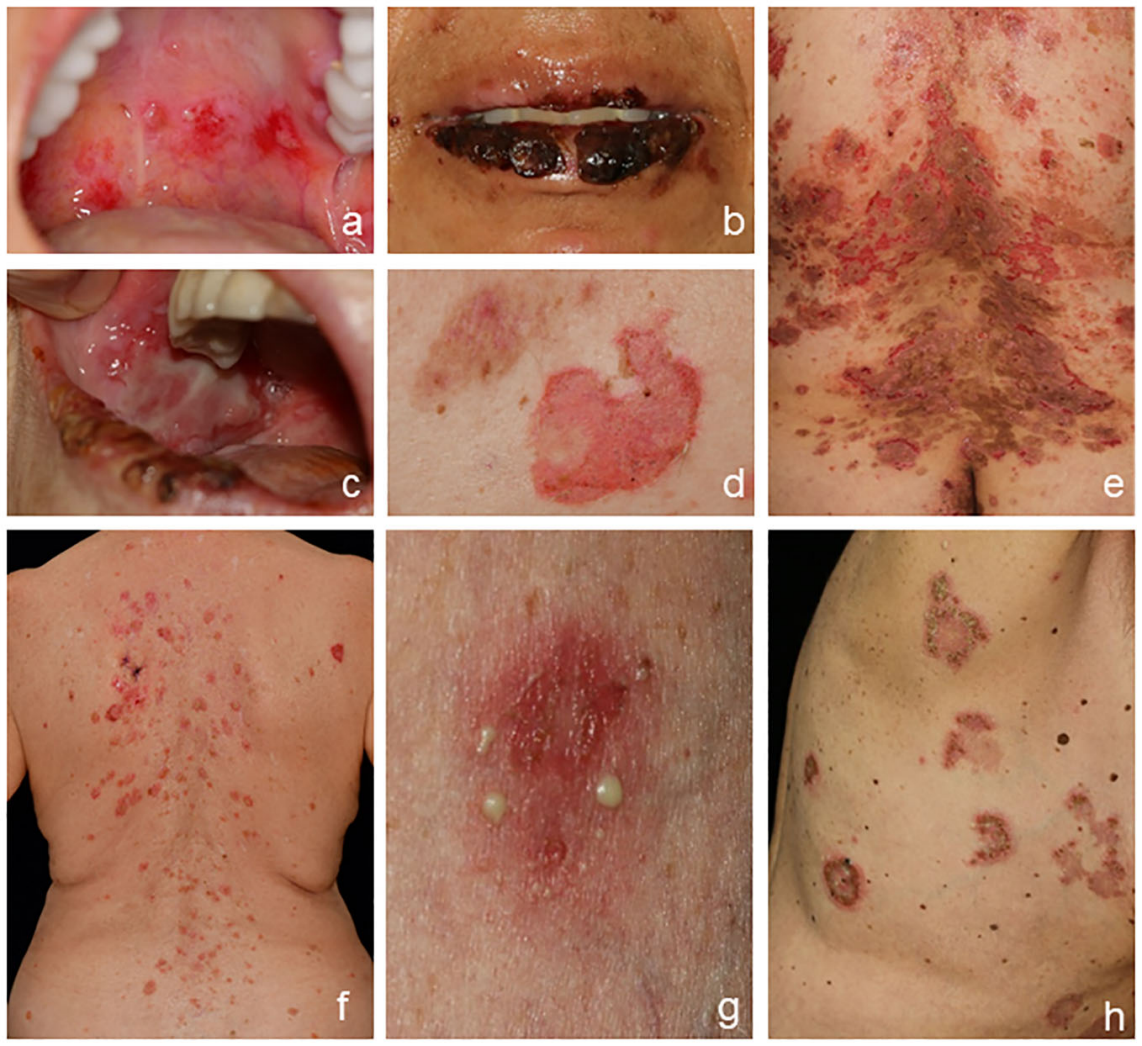
Clinical presentation of pemphigus. Classical presentation of pemphigus vulgaris with lesions of the oral mucosa, erosions on the soft palate **(A)**, crusts on the lips **(B)**, and erosions of the buccal mucosa **(C)**. Skin involvement shows erosions **(D, E)** and crusts with postinflammatory hyperpigmentation **(E)**. Pemphigus foliaceus with erythema and erosions predominantly in the seborrheic areas **(F)**. IgA pemphigus with pustules, erosions, and blisters **(G, H)**.

Pemphigus diseases are clinically characterized by flaccid blisters and erosions on the skin and/or surface-close mucous membranes. In pemphigus vulgaris (PV), most patients initially develop painful and sometimes bleeding erosions of the oral mucosa that can lead to difficulties in food intake and consequently to weight loss (**Figures 1A–C**). Oral lesions are often localized on the palatine and buccal mucosa, but other mucous membranes of the nose, pharynx, hypopharynx, esophagus, and genitals may be involved as the disease progresses. Additionally, erosions on the skin surrounded by erythema are common, while frank blistering is rare due to the fragility of the blisters resulting from the intraepidermal splitting (**Figures 1D–F**). Skin lesions show a positive Nikolsky sign, i.e., epidermal separation on tangential mechanical pressure, and are usually less pruritic in contrast to pemphigoid diseases (1–3, 27). Pemphigus foliaceus (PF) does not involve mucous membranes and solely presents on the skin with cutaneous “puff pastry-” or “cornflakes-like” exfoliation, erosions, crusts, and scales. Skin lesions are often found in the seborrheic sites including the face and scalp as well as the upper chest and back (**Figures 1G, H**). The rare IgA pemphigus is categorized into a subcorneal pustular dermatosis type and an intraepidermal neutrophilic dermatosis type. Both subgroups present with predominantly cutaneous and often pruritic lesions with pustules and blisters configured in an annular or circinate pattern with central crusting; mucous membranes are occasionally involved (28). Paraneoplastic pemphigus (PNP) clinically resembles PV with a more multiform presentation including flaccid or tense blisters and pustules as well as often severe ulcerating oral lesions typically affecting the lips and tongue (23, 29, 30).

**Figure 2:**
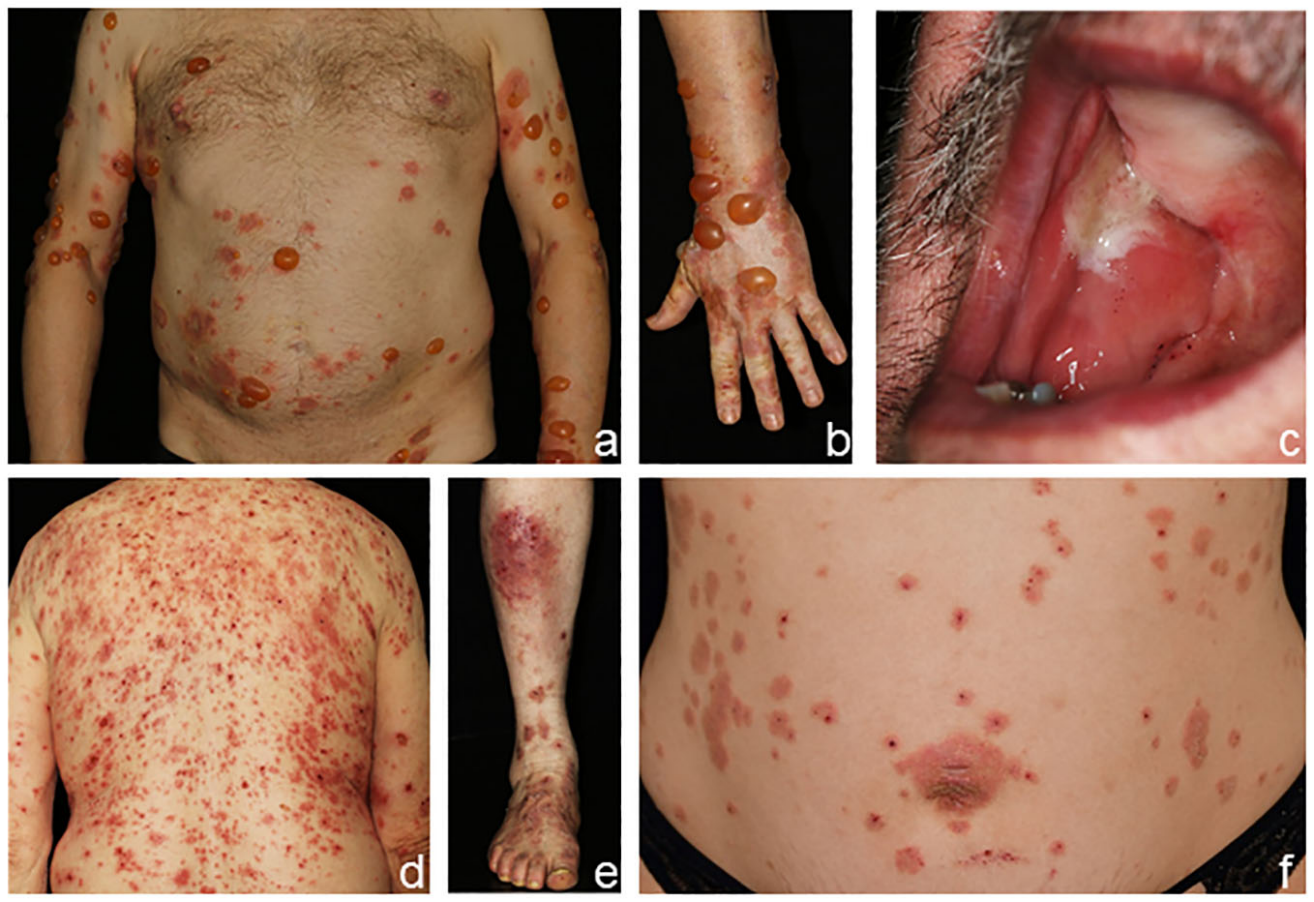
Clinical presentation of bullous pemphigoid **(A–E)** and pemphigoid gestationis **(F)**. Tense blisters and erythematous plaques on the trunk and arms **(A, B)**, fibrin-covered erosions of the buccal mucosa and palate **(C)**, and eczematous and urticarial lesions in bullous pemphigoid **(D, E)**. Pemphigoid gestationis in a pregnant woman with pruritic plaques and excoriations around the umbilical region **(F)**.

Bullous pemphigoid (BP) presents with intense pruritus and tense blisters of the skin and sometimes additionally mucous membranes (**Figures 2A–C**) (6, 31). In BP, an early pre-bullous stage can present with solely pruritus, eczema, or urticarious lesions (**Figures 2D, E**). With disease progression, tense blisters can form and persist for several days before eventual rupture leads to encrusted plaques and erosions (32, 33). In BP, mucous membranes are affected in 10%–20% of patients (34); however, mostly only mild oral lesions are seen. Pemphigoid gestationis clinically resembles the urticarial form of BP and is found in pregnant women in the second half of pregnancy or after delivery. Initially, in many patients, intensely pruritic plaques and, later, blisters form around the umbilical region and eventually disseminate across the trunk and extremities (**Figure 2F**) (6, 31, 35). The disease usually ceases within a few months but typically relapses during subsequent pregnancies.

**Figure 3:**
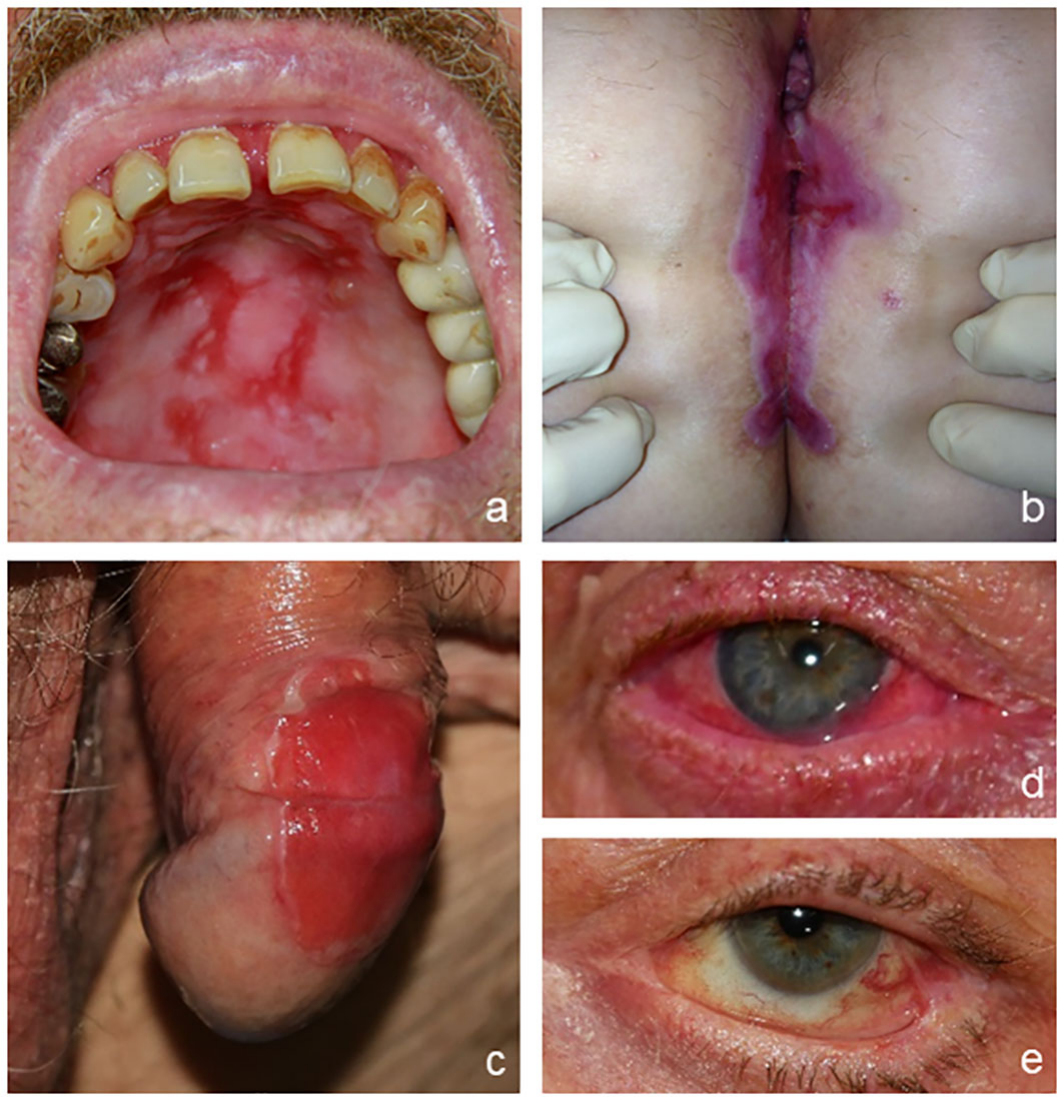
Clinical presentation of mucous membrane pemphigoid. Erosions on the hard palate **(A)**; vulva, perineal, and perianal regions **(B)**; and penis **(C)**. Conjunctival erythema **(D)** and symblepharon **(E)** as typical features of ocular involvement.

MMP is defined as pemphigoid disease with predominant mucosal involvement (36). The mucosa can be affected at various sites as well as the skin. The most commonly affected location is the oral mucosa with erosions and blisters appearing primarily at the gingival and buccal regions that can consequently lead to scarring (**Figures 3A**) (31, 37, 38). The second most frequently involved site is the ocular conjunctiva. Patients typically present with reddened and irritated eyes with conjunctivitis that can lead to scarring and the formation of symblephara, culminating in severe mutilations that can result in blindness (**Figures 3B, C**). The nasal mucosa, pharynx, larynx, trachea, esophagus, and genital mucosa can be involved and might result in dysfunctional scarring (**Figures 3D, E**) (39–41). Scarring, which occurs localized, mainly on the scalp, and may be associated with (subsequent) oral erosions, is referred to as Brunsting–Perry pemphigoid (42).

**Figure 4:**
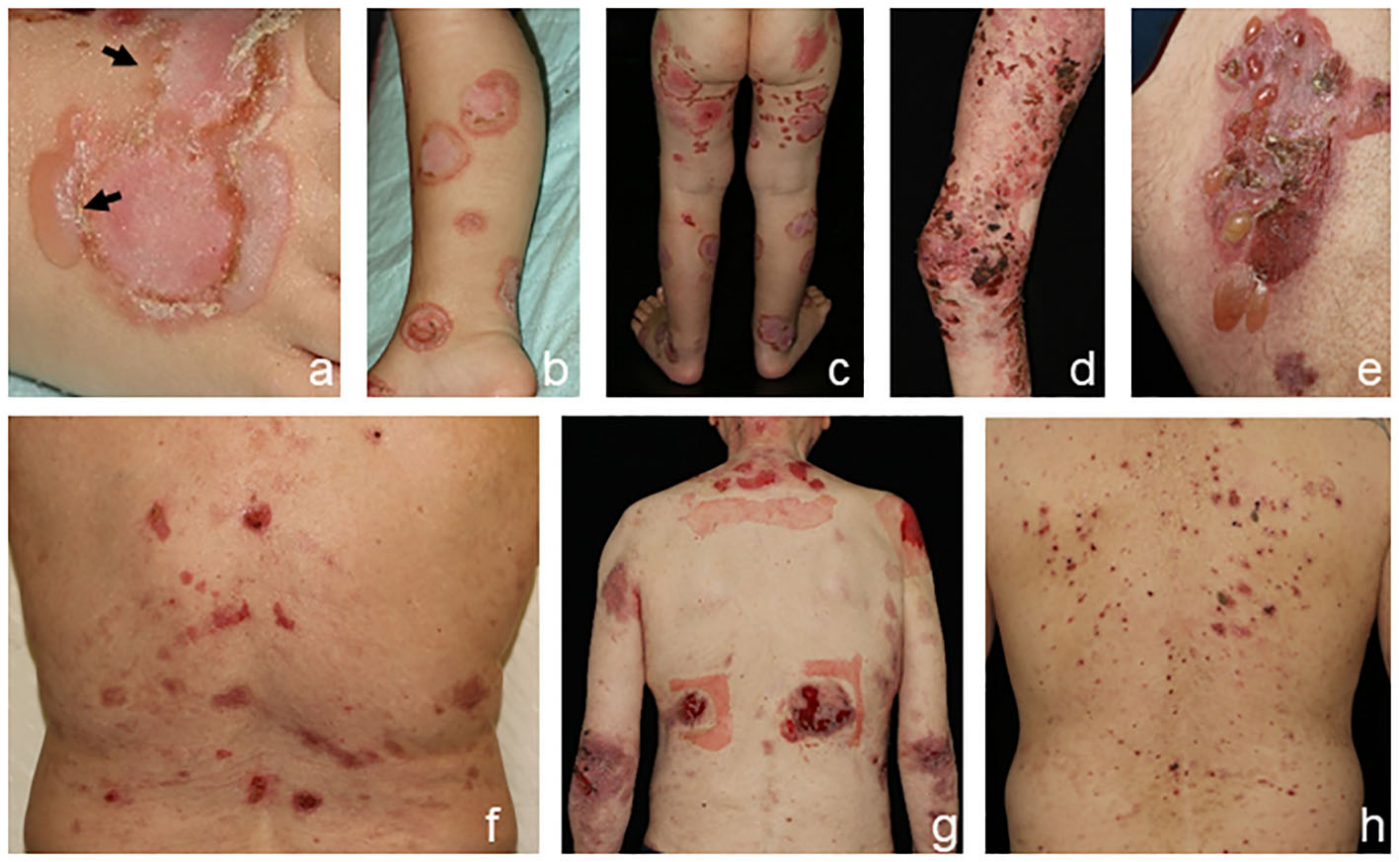
Clinical presentation of linear IgA disease (LAD), **(A–C)** anti-p200 pemphigoid, **(D, E)** and epidermolysis bullosa acquisita (EBA) **(F–H)**. LAD in a 4-year-old child with blisters along the edges of erythematous plaques (➔, “string-of-pearls”, **A**). Annular pattern of lesions on the legs **(B, C)**. Anti-p200 pemphigoid with pruritic urticarial plaques and tense blisters **(D, E)**. Inflammatory variant of EBA presenting with erosions and postinflammatory hyperpigmentation on the back **(F)**. Mechanobullous form of EBA with high skin fragility and erosions after the removal of Band-Aids **(G)**. IgA EBA manifesting with eczema, excoriated papules, and erythematous plaques **(H)**.

Linear IgA disease (LAD) presents with blisters arranged along the edges of round erythematous plaques in a typical annular pattern termed “string-of-pearls” (**Figures 4A–C**) (5). Additionally, oral, nasal, or genital lesions are often found. LAD is the most frequent AIBD in children and adolescents (18).

The clinical presentation of anti-p200 pemphigoid is diverse and mostly resembles BP. The LAD-like subtype is characterized by grouped blisters in an annular configuration. Palms and soles are frequently involved, whereas mucous membranes are affected in approximately 30%–40% of patients (**Figures 4D–F**), and an association with comorbid psoriasis, pronounced in Japanese patients, has been reported (43–46).

Epidermolysis bullosa acquisita (EBA) is clinically heterogeneous. In approximately one-third of patients, the mechanobullous (classical) form manifests as characterized by skin fragility and tense blisters forming upon minor traumata on exposed extensor skin locations without signs of inflammation. Healing is often associated with hyper-/hypopigmentation and scarring (**Figures 4G, H**) (6, 22). Nail dystrophy or scarring alopecia is frequently seen. In contrast, the inflammatory subtype manifests in two-thirds of patients and mimics the clinical features of BP or LAD. Clinical pictures of classical and inflammatory types of EBA can be present in the same patient, both simultaneously and sequentially. Mucous membranes are frequently involved, but when mucosal lesions predominate, MMP is diagnosed (22, 47).

Bullous systemic lupus erythematosus (SLE) is an AIBD occurring in SLE. The disease presents with eruptive evolution of tense bullae preferentially on sun-exposed skin with inflammatory erythematous plaques on the face, neck, upper trunk, and supraclavicular region, as well as axillary folds and oral and vulvar mucosa, but may erupt anywhere. The clinical phenotype is heterogeneous and can resemble other types of vesiculobullous diseases including BP, EBA, or dermatitis herpetiformis (48).

Rare variants of pemphigoid diseases include cicatricial pemphigoid characterized by scarring lesions without predominant mucosal involvement, IgM pemphigoid that usually manifests without frank blistering but with pruritic erythematous lesions, and lichen planus pemphigoides. In the latter pemphigoid disease, tense blisters arise also adjacent to lichen planus lesions. Furthermore, pemphigoid induced by orf virus infection has been described and linked to anti-laminin 332 autoantibodies (49–52).

Clinically distinct from pemphigoid diseases, subepidermal blistering in dermatitis herpetiformis leads to urticarial plaques, small vesicles, and papules arranged in a herpetiform pattern and located to the extensor skin surfaces, typically involving the buttocks, knees, and elbows. Dermatitis herpetiformis is characterized by intense pruritus and sometimes stinging sensations (8, 25, 53).

## Direct immunofluorescence microscopy

Direct IFM of a perilesional biopsy is the gold standard in the diagnosis of AIBDs. This method is based on the detection of tissue-bound autoantibodies in the skin or mucous membranes of patients. Superior sensitivity of approximately 91% and specificity of approximately 98% of direct IFM compared to other diagnostic methods in different autoimmune diseases have been demonstrated in various studies (54–58). Direct IFM is performed on cryosections of a perilesional biopsy of the skin or mucous membranes. The tissue specimen must be snap-frozen and stored at −20°C or preserved in isotonic NaCl solution or modified Michel’s medium until further processing (20).

**Figure 5:**
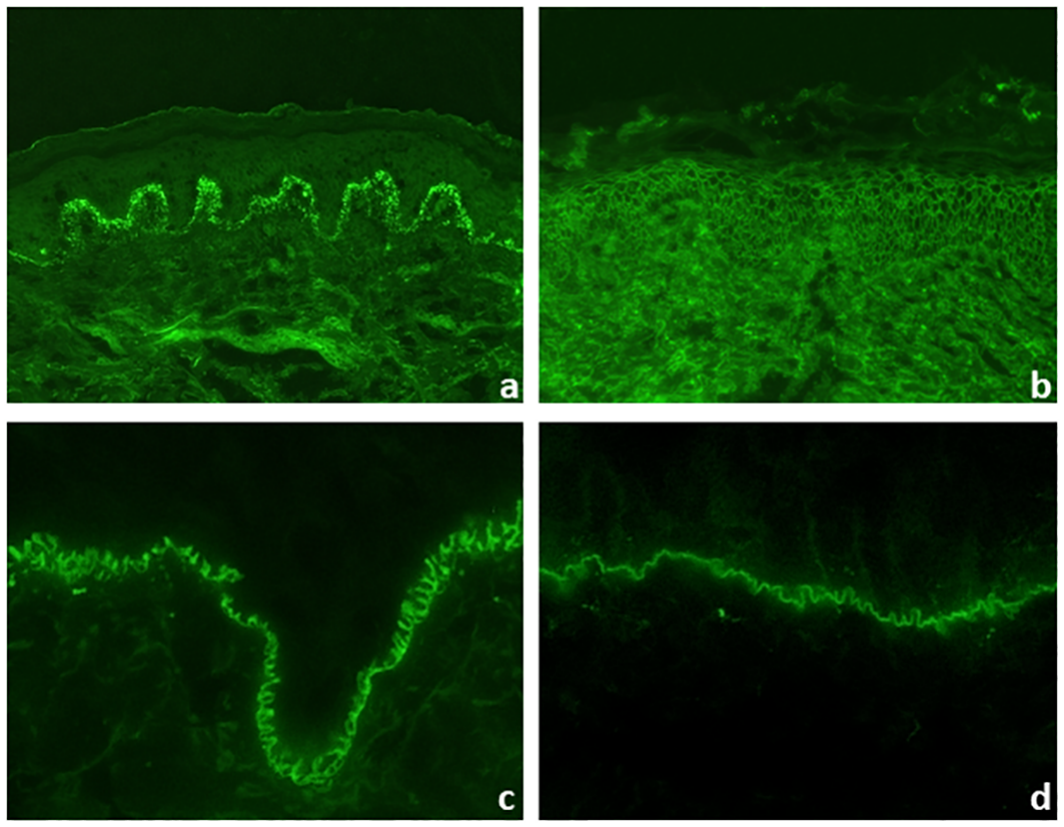
Direct immunofluorescence microscopy of perilesional biopsies. Granular deposits of IgA at the tips of dermal papillae and along the dermal–epidermal junction in dermatitis herpetiformis (×200 magnification) **(A)**. Intercellular deposits of IgG in the epidermis in pemphigus vulgaris (×200 magnification) **(B)**. In pemphigoid diseases, u- and n-serrated patterns of linear deposits at the dermal–epidermal junction can be distinguished. In epidermolysis bullosa acquisita, u-serrated pattern can be seen with arches closed at the bottom (×1,000 magnification) **(C)**. In all other pemphigoid diseases, n-serrated pattern is observed with arches closed at the top (×1,000 magnification) **(D)**.

Direct IFM allows the diagnosis of pemphigus and pemphigoid diseases as well as dermatitis herpetiformis by distinct binding patterns of immunoreactants. Direct IFM findings in dermatitis herpetiformis are mostly presented by granular deposits of IgA along the dermal–epidermal junction and/or at the tips of dermal papillae (**Figure 5A**) (25). In pemphigus diseases, IgG, IgA, and complement C3 bind to the desmosomes in the stratum spinosum of the epidermis/epithelium, forming intercellular net-like deposits (**Figure 5B**) (3, 20). In pemphigoid diseases, involved antigens are located at the dermal–epidermal junction, resulting in linear binding of IgG, IgA, and/or C3 and rarely of IgE and/or IgM at the BMZ (1, 6, 31). However, direct IFM only provides limited information on the involved target antigen. Discrimination between EBA and other pemphigoid diseases is possible using serration pattern analysis (59, 60). This is performed on thin (6-µm) cryosections of perilesional skin biopsies at 400–600-fold magnification using a conventional IF microscope. In EBA, immunoreactants demonstrate a “growing grass” or “upstanding arm” pattern with arches closed at the bottom, forming an “u” shape, which is termed u-serrated pattern (**Figure 5C**) (22). This pattern is pathognomonic for reactivity against type VII collagen, i.e., EBA and bullous SLE (54). In all other pemphigoid diseases, an n-serrated pattern with arches closed at the top is observed (**Figure 5D**) (61, 62). Differentiation between u- and n-serrated patterns only appeared feasible in approximately 75% of all samples and cannot be performed in mucosal biopsies (63, 64).

**Figure 6:**
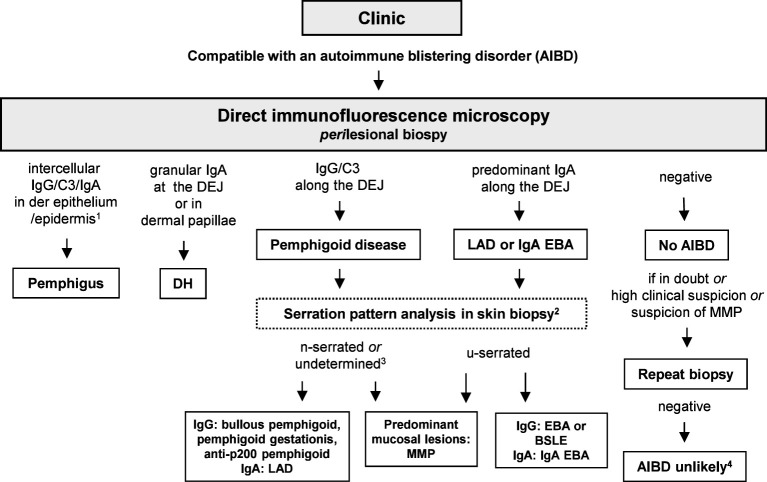
Diagnostic algorithm for direct immunofluorescence microscopy (IFM). 1, In paraneoplastic pemphigus, a combined pattern with anti-DEJ deposits may be present; 2, in mucosal biopsies, pattern analysis is not possible; 3, so far, in none of the undetermined cases, an EBA or BSLE has been diagnosed based on serum anti-type VII collagen IgG/IgA (64); 4, unless in ocular MMP, where direct IFM can be repeatedly negative; in these cases, diagnosis of MMP can be made based on the clinical picture and the clinical and histopathological exclusion of differential diagnoses (65, 68). BSLE, bullous systemic lupus erythematosus; EBA; epidermolysis bullosa acquisita; DH, dermatitis herpetiformis; DEJ, dermal–epidermal junction; LAD, linear IgA disease; MMP, mucous membrane pemphigoid.

Particularly in MMP, it has been shown that the diagnostic sensitivity can be greatly increased by taking a new biopsy and repeating direct IFM (65, 66). In oral biopsies of PV and MMP, a non-lesional biopsy is equally sensitive than a perilesional (67). An overview of the evaluation of direct IFM for AIBDs is shown in **Figure 6**.

## Serology

### Indirect immunofluorescence microscopy on tissue substrates

**Figure 7:**
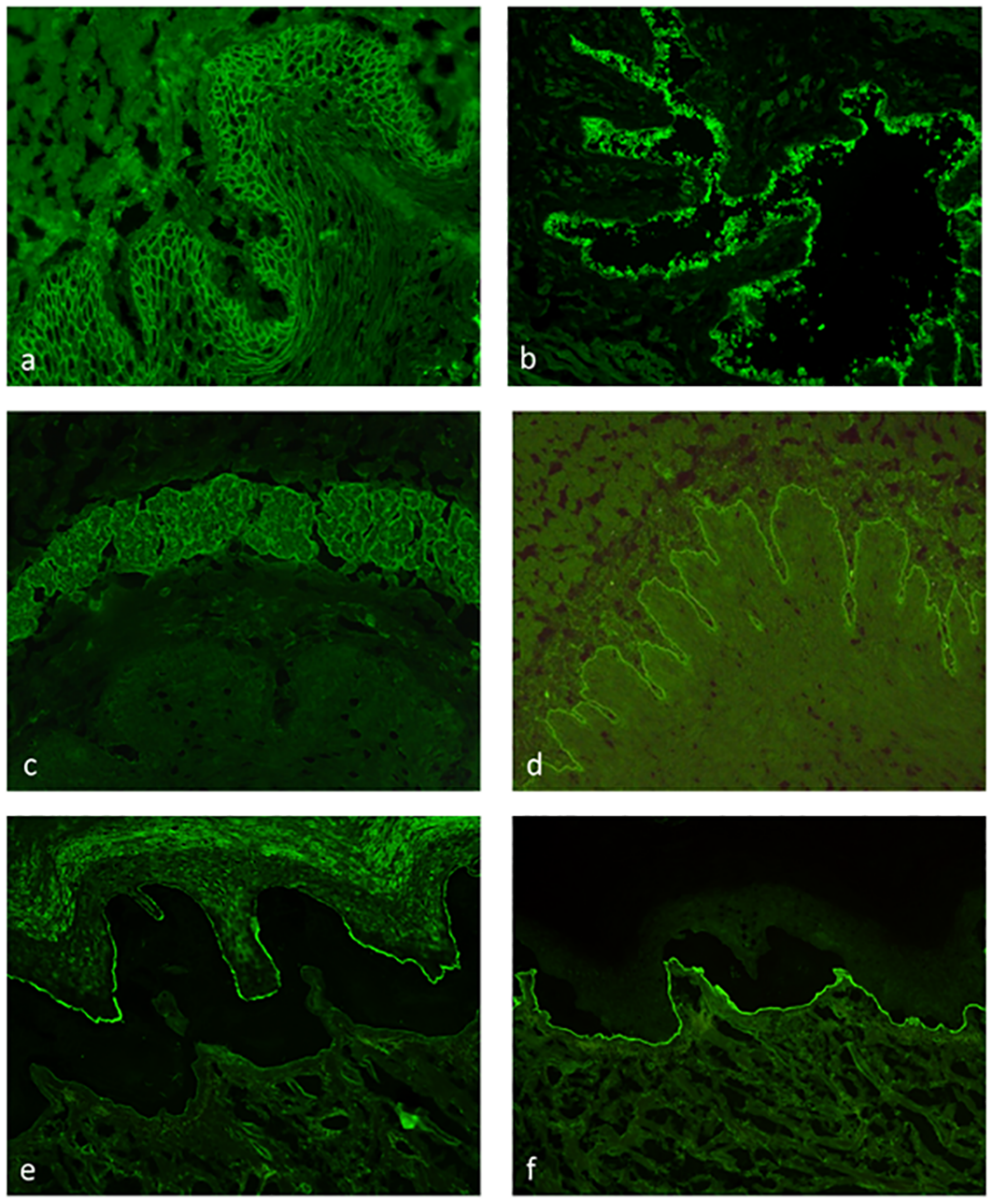
Indirect immunofluorescence on monkey esophagus, rat bladder, and human salt-split skin. Detection of IgG binding with an intercellular pattern in the epithelium of monkey esophagus pemphigus vulgaris **(A)**. IgG reactivity to rat bladder epithelium typical for paraneoplastic pemphigus **(B)**. IgA binding to the endomysium of monkey esophagus in dermatitis herpetiformis **(C)**. Linear IgG binding on monkey esophagus **(D)** and along the roof of the artificial blister of salt-split human skin **(E)** in a bullous pemphigoid patient. IgG reactivity along the blister floor of salt-split human skin, which can be seen in anti-laminin 332 mucous membrane pemphigoid, epidermolysis bullosa acquisita, and anti-p200 pemphigoid **(F)**.

**Figure 8:**
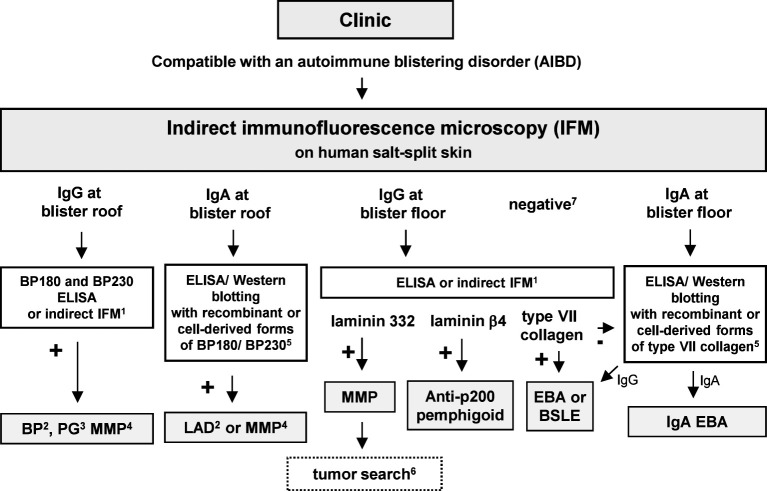
Diagnostic algorithm for serum autoantibodies in pemphigoid diseases. 1, Commercial assays are available; 2, with predominant skin lesions; 3, in pregnancy; 4, with predominant mucosal lesions; 5, in-house assays only available in specialized laboratories; 6, in approximately 25% of MMP patients with anti-laminin 32 reactivity, a malignancy was found; 7, on high clinical suspicion of BP, BP180 NC16A-specific ELISA/indirect IFM is recommended; on suspicion of MMP, additional serological tests in particular for IgG reactivity against laminin 332 are recommended; on suspicion of EBA, type VII collagen-specific ELISA/indirect IFM is recommended. BP, bullous pemphigoid; BSLE, bullous systemic lupus erythematosus; EBA; epidermolysis bullosa acquisita; DH, dermatitis herpetiformis; DEJ, dermal–epidermal junction; LAD, linear IgA disease; MMP, mucous membrane pemphigoid; PG, pemphigoid gestationis.

Indirect IFM on tissue substrates is a screening method for circulating autoantibodies in AIBDs (69). Here, tissue substrates were incubated with patient sera followed in a second step, with fluorescence-labeled anti-human-IgG/IgA/and sometimes IgM antibodies. Monkey esophagus is the most sensitive tissue substrate for the detection of autoantibodies in PV and PF, displaying an intercellular net-like fluorescence within the epithelium (**Figure 7A**). For autoantibodies in PF, guinea pig esophagus has been reported with an even higher sensitivity. In the case of paraneoplastic pemphigus, plakin autoantibodies can be best detected in monkey or rat bladder (**Figure 7B**). IgA reactivity to the endomysium of monkey esophagus is a diagnostic hallmark of dermatitis herpetiformis and is also positive in patients with celiac disease alone (25) (**Figure 7C**). Pemphigoid diseases can also be diagnosed by indirect IFM using a monkey esophagus showing linear anti-BMZ reactivity (**Figure 7D**) with a sensitivity of 60%–70% (69, 70) or normal human skin with a sensitivity of 80% (71). Sensitivity for pemphigoid autoantibodies is further increased by the use of human salt-split skin; i.e., normal human skin that is incubated with 1 M NaCl solution to induce artificial splitting within the lamina lucida (72, 73). Furthermore, NaCl-split skin allows the differentiation of pemphigoid autoantibodies in those with binding to the epidermal (against BP180 and BP230; **Figure 7E**) and dermal side (against laminin 332, p200 protein, and type VII collagen; **Figure 7F**). The diagnostic algorithm for indirect IFM on human salt-split skin is shown in **Figure 8**.

Since the main IgG subclasses in pemphigoid gestationis are IgG1 and IgG3, which are strongly complement activating, a complement source is added after incubating the serum on human salt-split skin, and by this, complement binding can be detected by a fluorescence-labeled anti-human-C3 antibody to visualize the respective autoantibody, previously termed “herpes gestationis factor” (74, 75).

### Enzyme-linked immunosorbent assay

Following the molecular identification of most target antigens in AIBDs, enzyme-linked immunosorbent assay (ELISA) based on the immunodominant parts of the recombinant antigens has become widely available for specific and sensitive detection of circulating IgG against desmoglein 1, desmoglein 3, BP180 NC16A, BP230, and type VII collagen (76–83). Serum levels of anti-desmogleins 1 and 3 IgG as well as anti-BP180 NC16A IgG and type VII collagen were shown to correlate with disease activity in most PV, PF, BP, and EBA patients, respectively (78, 79, 82, 84–86). Regular evaluations of serum autoantibody levels at follow-ups are thus recommended in current guidelines for these diseases (19–21, 87). To enable a parallel identification of different autoantibodies, multivariant ELISA has been introduced, compiling the different target antigens, i.e., desmoglein 1, desmoglein 3, BP180 NC16A, BP230, type VII collagen, and envoplakin, on a single ELISA plate (81).

Patients with dermatitis herpetiformis develop IgA antibodies against gliadin, endomysium, tissue-type transglutaminase (TG2), and epidermal transglutaminase (TG3). Various specific and sensitive ELISA for serum IgA and IgG reactivity against TG2, TG3, and deamidated gliadin-analogous fusion peptides are widely available (25, 88).

### Biochip-based indirect immunofluorescence microscopy

**Figure 9:**
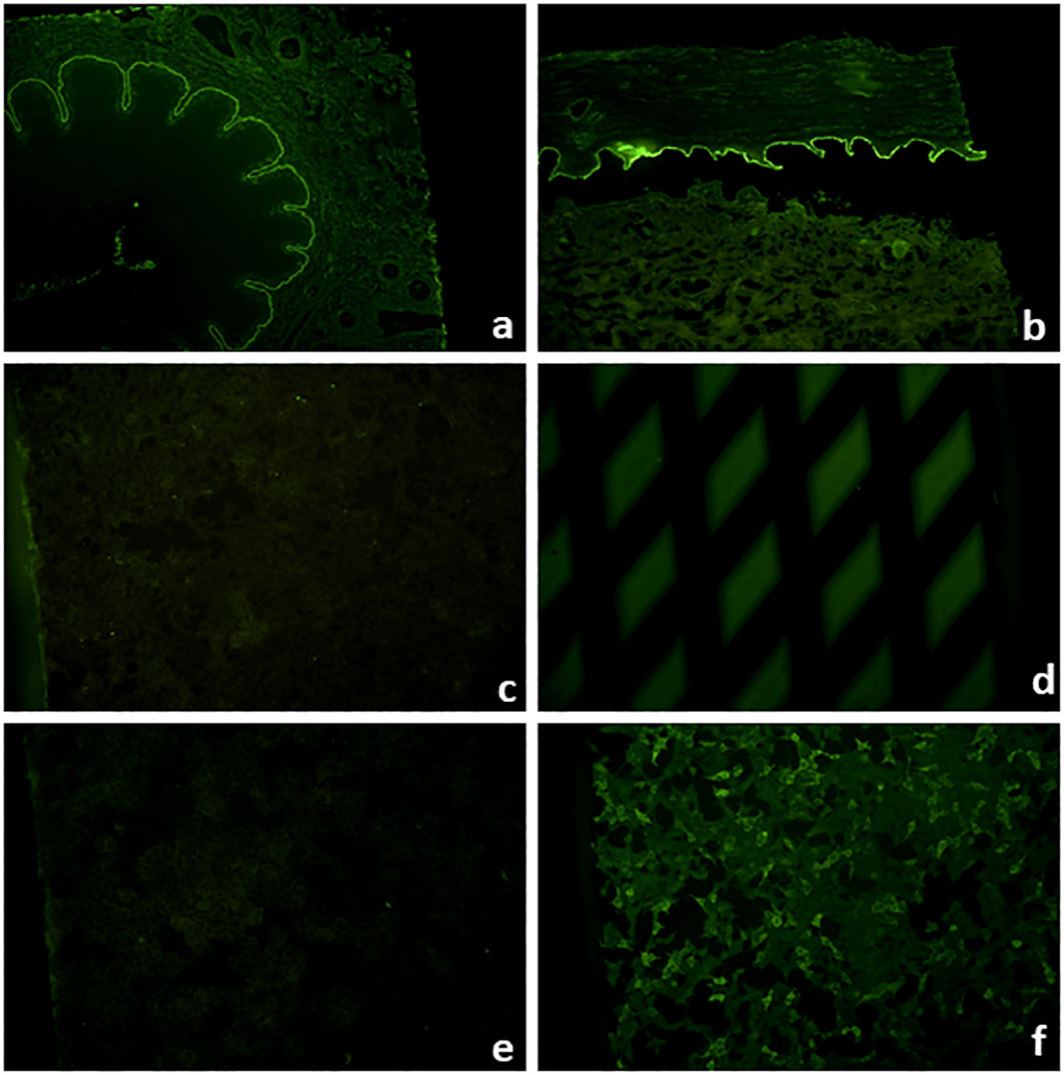
Biochip™ mosaic with six substrates that allow the serological diagnosis of approximately 90% of autoimmune blistering diseases when the clinical picture is known (89). Linear IgG binding on monkey esophagus **(A)**, primate salt-split skin blister roof **(B)**, positive IgG staining on recombinant BP180 NC16A **(D)**, and positive fluorescence of HEK293 cells transfected with BP230 **(F)**, while HEK293 cells transfected with desmogleins 1 **(C)** and 3 **(E)** show no fluorescence compatible with bullous pemphigoid.

Biochips™ are miniature substrates that can be assembled together in a so-called mosaic in a single incubation field of a laboratory slide. An individual Biochip™ can be composed of a tissue substrate, transfected cells expressing the recombinant target antigens on the cell surface (**Figure 9**), or directly the spotted recombinant target antigen. For AIBDs, different Biochip™ mosaics have been developed, and the most widely used is composed of six Biochips™, i.e., primate salt-split skin, primate esophagus, recombinant BP180 NC16A, and HEK293 cells transfected with the c-globular domain of BP230, desmoglein 1, and desmoglein 3 (89–96). This approach allows simultaneous testing of autoantibodies against several antigens in a time and serum-saving manner, as reactivity to several antigens can be tested in parallel. Its diagnostic accuracy has been shown to be comparable to that of standard multistep approaches and allows the serological diagnosis of more than 90% of AIBDs (89–94). For this Biochip™ mosaic, an automated reading and evaluation based on deep learning algorithm has recently been developed and will further facilitate and speed up the diagnostic process for AIBDs at least for laboratories with a high throughput (97).

**Figure 10:**
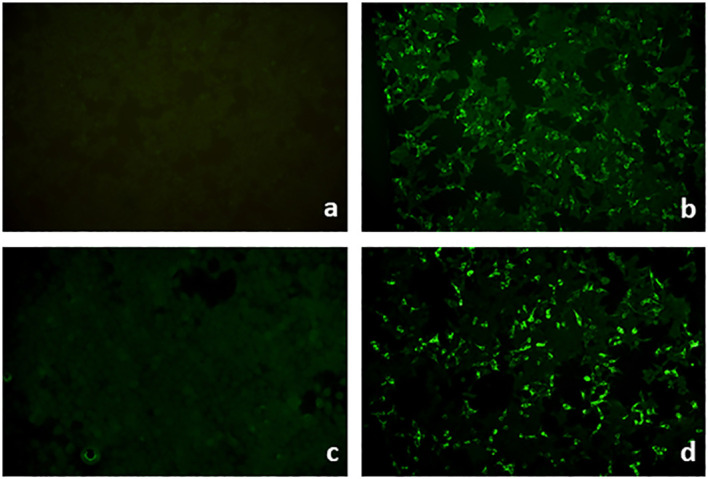
Biochips™ for serum IgG against laminin 332 and laminin β4. Biochips™ with HEK293 cells transfected with either the heterotrimer of laminin 332 **(A, B)** or laminin β4 **(C, D)**. IgG reactivity is seen in a patient with anti-laminin 332 mucous membrane pemphigoid **(B)** and a patient with anti-p200 pemphigoid **(D)**, while the corresponding negative controls show no reactivity **(A, C)**, respectively.

Further developments include Biochips™ with transfected HEK293 cells expressing desmocollins 1, 2, and 3; the laminin 332 heterotrimer (**Figures 10A, B**); the NC1 domain of type VII collagen; and most recently, laminin β4 (**Figures 10C, D**) (24, 98–103).

### In-house assays

**Figure 11:**
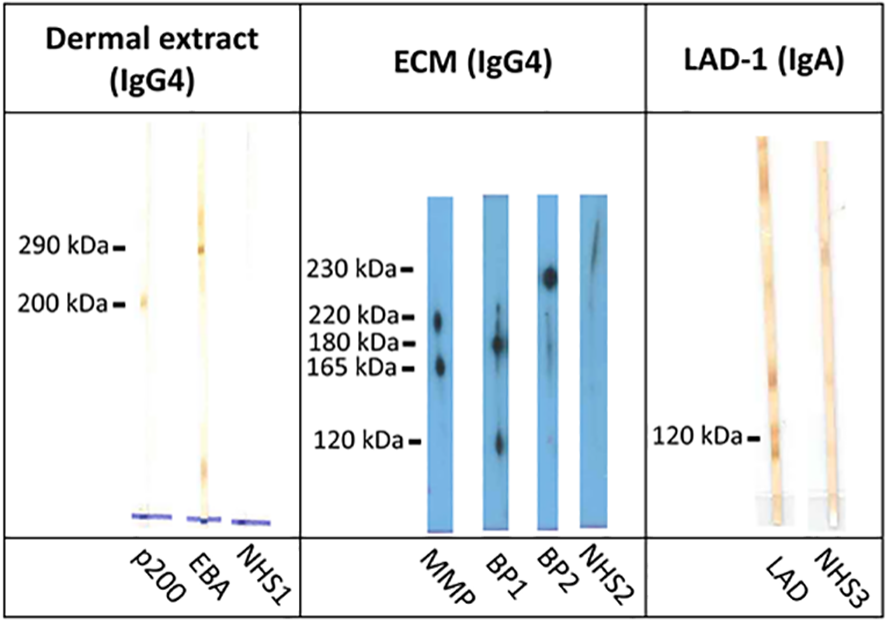
Immunoblotting with dermal extract, extracellular matrix of cultured human keratinocytes, and conditioned concentrated medium of cultured human keratinocytes. Immunoblot with extract of human dermis shows reactivity against the 200-kDa p200 protein in anti-p200 pemphigoid (p200) and the 290-kDa full-length type VII collagen in epidermolysis bullosa acquisita (EBA). IgG4 reactivity with the α3 chain of laminin 332 in mucous membrane pemphigoid (MMP; 220-kDa unprocessed and 165-kDa processed forms) as well as with BP180 (180-kDa full-length and 120-kDa processed forms) and with BP230 (230 kDa) in patients with bullous pemphigoid without reactivity against the immunodominant NC16A domain (BP1 and BP2) by immunoblotting with extracellular matrix of cultured human keratinocytes. Immunoblot with conditioned concentrated medium of cultured human keratinocytes for detection of IgA antibodies against the soluble ectodomain of BP180 [linear IgA disease antigen 1; LAD-1 in linear IgA diseases (LAD)]. Normal human sera (NHS1–3) were used as controls.

For the detection of some rare autoantibody specificities, no commercial tests are available. A few specialized laboratories have developed various in-house assays to overcome this problem. By using different cellular extracts or recombinant fragments of the target antigens and/or secondary antibodies for IgA reactivity, various additional autoantibodies can be detected by immunoblotting, ELISA, and immunoprecipitation. Until recently, the diagnosis of anti-p200 protein was based on the detection of IgG reactivity against the 200-kDa p200 protein in the extract of the human dermis or epidermis by immunoblotting (104–106) (**Figure 11**). The same assay can be used to detect IgG against the 290-kDa full-length type VII collagen (**Figure 11**). Alternatively, recombinant laminin γ1 applied by immunoblotting allowed the detection of serum autoantibodies in 70%–90% of anti-p200 pemphigoid patients (44, 45, 105, 107). Instead of recombinant laminin γ1, recombinant laminin trimers containing this subunit have been applied (71, 108–110).

The extracellular matrix of cultured human keratinocytes is used to detect antibodies against laminin 332 (111). In addition, reactivity against BP180 and BP230 can also be seen in this test (**Figure 11**). IgA autoantibodies against the LAD antigen-1 (LAD-1), which is the cell-derived soluble ectodomain of BP180 and primary target antigen in LAD, can be detected by immunoblotting with conditioned concentrated supernatant of cultured keratinocytes (112, 113). LAD-1 is also recognized by a subgroup of patients with BP (IgG) and MMP (IgG and/or IgA) (**Figure 11**). Furthermore, various recombinant fragments of the BP180 C-terminus were applied by immunoblotting and ELISA to describe autoantibodies in BP patients without anti-NC16A reactivity and in MMP (114). For IgA reactivity against the NC16A domain of BP180 in LAD and the NC1-domain of type VII collagen as well as the full-length type VII collagen in IgA EBA, immunoblotting can also be employed (115–117).

A dozen methods for the detection of anti-laminin 332 serum antibodies in MMP have been described including immunoprecipitation, immunoblotting, and ELISA with recombinant or cell-derived forms of this molecule (118–120). Recently, antibodies against laminin 332 have been determined by the so-called keratinocyte footprint assay, i.e., an indirect IFM where normal human keratinocytes are grown on glass coverslips and anti-laminin 332 IgG binds to the extracellular matrix in a characteristic pattern after removal of keratinocytes from the coverslips (121).

In paraneoplastic pemphigus, detection of autoantibodies against periplakin, epiplakin, plectin, desmocollin, and α2-macroglobulin-like 1 has been achieved by various sophisticated in-house assays (3, 29, 100, 122–126). So far, only a few studies have shown reactivity against α6β4 integrin in a subgroup of patients with MMP (127, 128).

ELISA detected IgE autoantibodies against BP180 NC16A in 40%–70% of BP patients, but without increasing diagnostic sensitivity (129–133). In the case of MMP and the mucosal variant of PV, BP180 NC16A and desmoglein 3-specific ELISA using salivary samples have been described to detect IgA/IgG reactivity (134, 135).

## Histopathology

For histopathology of AIBDs, lesional skin biopsies were investigated. Here, a differentiation between intraepidermal split formation with acantholysis in pemphigus diseases and subepidermal splitting in pemphigoid diseases and dermatitis herpetiformis can be achieved (136). Pemphigus samples present with subcorneal splitting in PF and PV, with acantholysis and suprabasal splitting, typically in a so-called tombstone pattern in PV, i.e., with remaining keratinocytes attached to the BMZ (2, 3). In paraneoplastic pemphigus, additional dyskeratosis, degeneration of the BMZ, and lichenoid lymphocytic infiltrates of the adjacent upper dermis can be seen (23). In contrast, IgA pemphigus shows subcorneal clefts with neutrophilic infiltrates and, if captured, subcorneal acantholysis (28). In pemphigoid diseases, subepidermal blister formation accompanied by an eosinophilic infiltrate is typical, but split formation might be lacking in pemphigoid gestationis and non-bullous forms of BP (137). Even in BP alone, lymphocyte-rich eosinophil-dominated and neutrophil-rich patterns can be observed (138). Classical patterns of LAD and dermatitis herpetiformis may present with neutrophil-dominated infiltrate along the dermoepidermal junction and in the dermal papillae forming microabscesses. Accompanying eosinophils and subepidermal blister formation may be present (139, 140). However, a clear differentiation based on histopathology alone is not feasible in pemphigoid disorders and dermatitis herpetiformis (53, 141, 142).

In pemphigoid diseases, staining for C3d/C4d reveals linear deposits at the BMZ (143–146). However, this method is hampered by its low sensitivity and specificity, most likely due to the lesional biopsy that favors unspecific labeling along the degraded BMZ and the degradation of immunoreactants by activated tissue proteases. As such, this method is only suitable in individual cases when no direct IFM and serology are available (147).

In summary, lesional histopathology is still recommended in every patient with suspicion of an AIBD (19, 20, 25, 29, 65, 68, 148). Its true value lies in its use to propose differential diagnoses when direct IFM and serology are unreactive. However, profound knowledge of the histopathological spectra of AIBDs is essential for every (dermato-) pathologist to recommend or initiate direct IFM and serology when such a disorder is suspected.

## Diagnostic criteria

### Pemphigus vulgaris and pemphigus foliaceus

The diagnosis of PV and PF is based on refs. (19, 21)

- clinical appearance with flaccid blisters erosions and
- intercellular net-like deposits of IgG/C3 in the epithelium in direct IFM and/or
- intercellular fluorescence on the epithelium of monkey esophagus in indirect IF and/or
- detection of anti-desmoglein 1 IgG (PF), anti-desmoglein 3 IgG (mucosal PV), or both (mucocutaneous PV) and/or
- suprabasal (PV) or subcorneal splitting (PF) with acantholysis in histopathology

Direct IFM establishes the diagnosis of a pemphigus disease by intercellular (sometimes granular appearing) epithelial staining. Indirect IFM testing on monkey esophagus as the most sensitive substrate uncovers circulating autoantibodies that cause intercellular staining of the epithelium (1–3). However, intercellular reactivity on monkey esophagus alone is not sufficient to establish the diagnosis of pemphigus, as it may be unspecific, e.g., by cross-reacting anti-blood group autoantibodies. Identification of the target antigens is performed by either ELISA or indirect IF-based Biochip™ technology (78, 79, 149). Levels of anti-desmoglein 1 and 3 serum IgG measured by ELISA usually correlate with disease severity and have been established to monitor the course of the disease as recommended by national and international guidelines to guide treatment decisions (20, 21, 78, 79).

If direct IFM is not available, detection of medium to high levels of circulating anti-desmoglein antibodies, compatible histological appearance, and a typical clinical presentation may suffice for diagnosis (20).

### IgA pemphigus

The diagnosis of IgA pemphigus can be established by

- clinical appearance with pustules and
- intercellular net-like deposits of IgA/C3 in the epithelium by direct IFM and/or
- intercellular fluorescence with IgA on the epithelium of monkey esophagus in indirect IFM and/or
- detection of anti-desmoglein 1 and/or 3 and/or anti-desmocollin IgA autoantibodies and/or
- subcorneal neutrophilic infiltrate with vesicles in histopathology

Autoantibodies of patients with the rare IgA pemphigus target desmocollins 1, 2, or 3 or desmogleins 1 and 3. Circulating IgA antibodies may be detected by indirect IFM on monkey esophagus and in-house Biochips™ or ELISA using the recombinant forms of these antigens (28, 100, 150).

Unlike PF and PV, lesional histopathology reveals marked infiltration with neutrophils, which causes the formation of pustules in initially appearing vesicles. In a perilesional skin biopsy, IgA deposits are seen with an intercellular pattern in the epidermis by direct IFM (21, 28, 151).

### Paraneoplastic pemphigus

The diagnosis of paraneoplastic pemphigus can be established by

- association with a malignancy
- clinical appearance with severe stomatitis and lip involvement
- intercellular net-like and additional linear deposits of IgG/IgA/C3 in direct IFM and/or
- IgG against urothelium by indirect IFM on monkey/rat bladder and/or
- detection of anti-envoplakin IgG by ELISA and/or
- detection of anti-plakin and/or anti-α2 macroglobulin like 1 autoantibodies and/or
- acantholysis, dyskeratosis, vascular degeneration of the BMZ, and lichenoid infiltrates in the upper dermis by lesional histopathology

Paraneoplastic pemphigus commonly leads to severe stomatitis including erosions on the tongue and lips. An underlying malignant disease, often hematological malignancies, thymoma, or Castleman disease, is a prerequisite for the diagnosis, but in some patients, it only manifests during the course of the disease. Direct IFM often uncovers linear IgG deposits at the dermal–epidermal junction in addition to a pemphigus-typical epidermal/epithelial intercellular distribution (2, 3, 21, 23). Histopathologically, lesions usually show acantholysis, dyskeratosis, vascular degeneration of the BMZ, and lichenoid infiltrates in the upper dermis (20, 23, 65). A broad variety of specific IgG antibodies may be found in serum, frequently directed against different plakins but also plectin, α2-macroglobulin-like 1, desmocollins 1–3, desmoglein 3, and BP180 (20, 21, 23, 30). The most relevant serological tests are indirect IM on monkey/rat bladder and the widely available envoplakin-specific ELISA (29). Additional in-house assays for the detection of autoantibodies periplakin, desmoplakin I/II, epiplakin, plectin, α2 macroglobulin like 1, and desmocollins 1, 2, and 3 are available in specialized laboratories.

### Bullous pemphigoid

The diagnosis of BP is based on refs. (19, 21)

- the clinical picture with blisters and erosions and/or urticarial plaques and erythema predominantly at the skin and
- linear deposits of IgG/C3 and sometimes additionally IgA in direct IFM and/or
- blister roof staining on salt-split skin by indirect IFM and/or
- detection of BP180/BP230 IgG by ELISA or indirect IFM and/or
- subepidermal blisters with eosinophilic–lymphocytic or neutrophilic infiltrates by lesional histopathology

By direct IFM linear depositions of IgG, C3 and sometimes additional IgA and IgM at the BMZ in an n-serrated pattern can be seen (1, 6, 60, 152). A further distinction from other AIBDs is required by serology, which displays epidermal binding of predominant IgG in salt-split skin and/or reactivity to recombinant BP180 NC16A and/or BP230 by ELISA or indirect IFM (1, 6) (**Table 1**).

**Table 1:** Autoantibody specificities and diagnostically relevant clinical signs.

Disease	Target antigen	Clinical signs of diagnostic relevance*
Pemphigus vulgaris	***Dsg 3***, *Dsg 1*	Erosions and flaccid blisters of mucous membranes, ± flaccid blisters and erosions on skin
Pemphigus foliaceus	***Dsg 1***	Flaccid blisters, erosions, scaling; no involvement of mucous membranes
Paraneoplastic pemphigus	***Dsg 3***, ***envoplakin***, periplakin, desmoplakin I/II, α 2 macroglobulin-like 1, plectin, epiplakin, Dsc 1, 2, 3	Severe stomatitis, erosions of lips, neoplasia (hematological malignancies, thymoma, Castleman disease)
IgA pemphigus	**Dsc 1–3** Dsg 1, Dsg 3	Mostly pustules and erosions
Bullous pemphigoid	***BP180 NC16A***, *BP230*	Tense blisters, erosions, urticarial plaques, severe pruritus, old age (>75 years)
Mucous membrane pemphigoid	**Non-BP180 NC16A (different epitopes)**, *BP180 NC16A*, ***laminin 332***, *BP230*, α6β4 integrin	Predominant mucosal involvement; oral and ocular mucosae are most frequently affected
Linear IgA disease	**LAD-1**, BP230 (IgA)	Tense blisters/vesicles, erosions, annular orientation of lesions (“string of pearls”)
Pemphigoid gestationis	***BP180 NC16A***, *BP230*	Erythema, papules, rarely vesicles, intense pruritus; pregnancy or post-partum period
Anti-p200 pemphigoid	***Laminin β4***, laminin γ1	Tense blisters, erosions
Epidermolysis bullosa acquisita	***Type VII collagen***	Mechanobullous and inflammatory variant (resembling BP or LAD)
Bullous systemic lupus erythematosus	*Type I:* ***type VII collagen*** type II*: BP180*, *BP230*, *laminin 332*	SLE present; tense blisters, erosions
Lichen planus pemphigoides	***BP180 NC16A***, *BP230*	Tense blisters independent of lichen planus lesions
Cicatricial pemphigoid	*BP180*, *BP230*, *laminin 332*	Skin blisters and erosions that heal with scarring and/or milia formation
IgM pemphigoid	BP180 (IgM)	Erythema and urticaria, excoriated papules/plaques, and lichenification
Dermatitis herpetiformis	***Epidermal TG***, ***tissue-type TG***, ***endomysium*** (IgA)	Severe pruritus, vesicles, erythematous excoriated papules on extremities and the gluteal region

Main target antigens are shown in bold; target antigens in italics indicate the wide availability of sensitive and specific assays.

Dsg, desmoglein; Dsc, desmocollin; TG, transglutaminase; LAD, linear IgA disease; BP, bullous pemphigoid; SLE, systemic lupus erythematosus.

*If not stated otherwise, skin involvement is predominant.

In case of epidermal binding in salt-split skin but no reactivity with the immunodominant NC16A region of BP180 or with BP230, other epitopes of BP180 might be targeted like the soluble cell-derived ectodomain or the C-terminal part of BP180. If no blister formation is present clinically, the European guideline recommends diagnosis based on compatible direct IFM findings and binding of circulating IgG to the epidermal side of salt-split skin by indirect IFM (19). In case of negative or unavailable direct IFM, diagnosis is concluded in case of a compatible clinical picture and consistent histology presenting with subepidermal blisters and eosinophilic infiltrates combined with serum IgG against BP180, BP230, and/or the epidermal side of salt-split skin by indirect IFM (19, 21). In the case of a classical clinical picture, the detection of serum IgG against BP180 NC16A in levels greater than threefold of the lower detection limit of the ELISA is sufficient for the diagnosis of BP (148).

### Mucous membrane pemphigoid

To diagnose MMP, a clinical presentation of erosions or blisters predominantly at mucous membranes is required. However, affection of mucous membranes as well as the targeted autoantigens are highly variable. Establishing the diagnosis is thus based on the following refs. (41, 65, 149):

- predominant mucosal blisters and/or erosions and
- linear deposits of IgG/IgA/C3 in direct IFM and/or
- linear staining at either blister roof or blister floor of salt-split skin by indirect IFM and/or
- serum reactivity against BP180, laminin 332, and type VII collagen

Diagnostics is further complicated by a lower positive rate of direct IFM samples ranging from 60% to 90% (38, 39, 65, 153). This issue can be circumvented in part by testing additional biopsies (66). As such, guidelines recommend in case of an initial negative direct IFM to repeat the testing with at least one additional biopsy (65, 68).

Serological diagnosis for MMP includes indirect IFM on salt-split skin for both IgA and IgG autoantibodies, which direct to subsequent assays for autoantibodies binding to the epidermal side (i.e., BP180 and BP230) or dermal side (i.e., laminin 332 and type VII collagen) (38, 65, 153, 154) (**Table 1**). Typically, first ELISA and Biochip™ analyses are prompted, followed by immunoblot-specific in-house assays for more rare target antigens (37, 38) (**Figure 8**). In case of negative findings on salt-split skin, ELISA/immunoblotting or Biochip™ techniques should be employed following the expected frequency of autoantibodies directed against targeted structures (39). However, low frequencies of circulating autoantibodies, in particular in ocular MMP, pose a challenge to serologic diagnostics (38, 65, 153, 154). The diagnosis of ocular MMP can be established without positive findings by serology and direct if other causes of cicatrizing conjunctivitis have been ruled out both clinically and histopathologically (65).

Of note, anti-laminin 332 reactivity warrants a tumor search since up to 30% of MMP patients with laminin 332-specific antibodies develop a malignancy (24, 38, 39, 65). As such, assaying for anti-laminin 332 reactivity is recommended in all newly diagnosed patients with MMP without clear antibodies against BP180 or type VII collagen and in case of initially negative reactivity, also during the course of the disease (65, 68).

Antibodies against α6β4 integrin have been described in MMP (127, 128). Following a systematic literature search, the recent S3 guideline has not recommended testing for anti-α6β4 integrin until further data are available (65).

### Pemphigoid gestationis

Gestational pemphigoid is a rare condition in pregnancy and needs to be differentiated from other pruritic eruptions in pregnancy. The diagnosis is based on the following ref. (35):

- compatible clinical picture with urticarial paraumbilical plaques and/or vesicles and in pregnancy or post partum period
- linear deposits of C3 and sometimes additional IgG in direct IFM and/or
- positive complement binding test and/or
- blister roof staining on salt-split skin by indirect IFM and/or
- reactivity with BP180 NC16A in antigen target-specific assays

Histopathology shows an eosinophilic infiltrate but is often unspecific, as the split formation is initially mostly lacking (155, 156). By direct IFM, mainly C3 deposits can be seen along the dermal–epidermal junction in a linear pattern, and only approximately one-third of patients show concomitant IgG deposition (155). Similarly, standard indirect IFM detecting IgG and IgA autoantibodies may be negative in 75% of patients. Adding a complement source (also referred to as complement binding test) may yield positive results of indirect IFM in most sera (155, 156). IgG-autoantibodies are mainly directed against the NC16A domain of BP180 and are mainly constituted of the IgG1 and 3 complement activating subclasses (75). Reactivity against recombinant BP180 NC16A can be detected by ELISA or indirect IFM in nearly all patients, and serum anti-BP180 NC16A levels correlate with disease activity (92, 156–159).

### Linear IgA disease

LAD is defined by predominant IgA autoantibodies against proteins of the BMZ. The diagnosis relies on the following ref. (51):

- clinical picture with vesicles and blisters frequently in an annular pattern with vesicles at the edge of lesions and
- predominantly IgA deposits along the BMZ by direct IFM and/or
- linear staining of blister roof or, more rarely, of floor on salt-split skin with predominantly IgA and/or
- IgA reactivity with LAD-1 or another epitope on BP180 and/or
- subepidermal splitting with neutrophils along the BMZ and in dermal papillae in lesional histopathology

By direct IFM next to the predominant IgA deposition, reactivity of C3 and to a lesser extent IgG and IgM can be seen (160). Circulating IgA autoantibodies usually bind at the blister roof of salt-split skin, where the main target antigen, the soluble ectodomain of BP180 (LAD-1), is situated (6, 161). In the case of staining with IgA at the blister floor of salt-split skin, the target antigen may be type VII collagen; here, the term IgA EBA has been proposed (162), and the European guideline, which is currently underway, will adopt this differentiation between LAD and, in case of predominant IgA reactivity against type VII collagen, IgA EBA. Histopathology is very similar to dermatitis herpetiformis with neutrophilic infiltrate along the dermal–epidermal junction and in the dermal papillae forming microabscesses. Accompanying eosinophils and subepidermal blister formation may be present (139, 163).

### Anti-p200 pemphigoid

Anti-p200 pemphigoid is the most recently recognized AIBD (106). It is defined by refs. (44, 45)

- predominant skin lesions with involvement of extremities, often the palms and soles, and
- linear deposits of IgG/C3 by direct IFM and/or
- blister floor staining on salt-split skin by indirect IFM with IgG and/or
- reactivity with the 200-kDa p200 protein by immunoblotting with dermal extract or
- reactivity with laminin β4 and/or laminin γ1
- subepidermal blistering with neutrophilic or mixed infiltrate in lesional histopathology

While by direct IFM and histopathology anti-p200 pemphigoid mainly resembles BP, its dermal reactivity on salt-split skin distinguishes it from BP (43, 45). Direct IFM serration pattern is n-serrated, thus excluding EBA as a differential diagnosis (60, 164). For exact diagnosis, deciphering the target antigen is required. Traditionally, this is performed by immunoblotting with dermal or epidermal extract (104, 106). Subsequently, laminin γ1 has been described as a target antigen and is recognized by 70%–90% of patients (45, 105, 165). Assays for the detection of anti-p200 and anti-laminin γ1 have only been available in specialized laboratories. Since the pathogenic relevance of anti-laminin γ1 IgG could not be shown *in vitro* and *in vivo*, it has been hypothesized that the true autoantigen of this disease is still to be discovered (166, 167). In fact, most recently, laminin β4 has been reported as a target antigen recognized by nearly all patients (168, 169). A Biochip™-based indirect IFM assay for the detection of circulating laminin β4 is widely available (169). The pathogenic potential of anti-laminin β4 IgG is currently being investigated.

### Epidermolysis bullosa acquisita

EBA is caused by autoantibodies to type VII collagen, the major component of anchoring fibrils. Clinically and histologically EBA can resemble other pemphigoid diseases, so diagnosis of EBA based only on clinical picture and histology alone is not possible, in particular in the inflammatory variant.

Diagnosis of EBA is based on (170)

- u-serration pattern by direct IFM
- detection of circulating anti-type VII collagen autoantibodies by ELISA, indirect IFM (Biochip™), or immunoblot with dermal extract or the recombinant NC1 domain
- indirect IFM on type VII collagen-deficient skin (only applicable when serum antibodies are reactive against human/primate skin)
- direct immunoelectron microscopy
- fluorescence overlay antigen mapping (FOAM)

Direct IFM of perilesional biopsy demonstrates linear deposits of IgG, IgA, or complement along the dermal–epidermal junction. By close inspection of 4–6-µm-thick sections at ×400–×600 magnification, the characteristic u-serrated pattern can be observed (**Figure 5C**). This pattern is pathognomonic for skin-bound antibodies to type VII collagen and resembles “growing grass”. However, differentiation between u- and n-serrated patterns is not possible in approximately one-quarter of samples as well as in mucosal biopsies (59, 60, 64).

Immunoelectron microscopy, which used to be the diagnostic gold standard for EBA, allows the detection of autoantibody deposits in anchoring fibrils in the sublamina densa, which is typical for EBA or bullous systemic lupus erythematosus (BSLE) (171, 172). Unfortunately, this method requires the processing of fresh biopsies and is only performed in a few laboratories in the world. Another technique that could distinguish EBA from other pemphigoid diseases is FOAM. This method is based on the coincubation of perilesional biopsy with antibodies against other antigens, such as BP180, laminin 332, and type VII collagen. Overlay of different pictures allows detection of colocalization of autoantibodies (173, 174).

While diagnosing AIBDs by detecting circulating autoantibodies has become a major diagnostic approach (6, 69, 175), serological diagnosis in EBA is limited by the relatively low rate of circulating antibodies in approximately 60% of patients (54, 55). Antibodies to type VII collagen bind along the BMZ on monkey esophagus and label the floor of the artificial blister in salt-split skin (**Figure 7F**), similar to anti-p200 and anti-laminin 332 antibodies. Usage of type VII collagen-deficient skin as the substrate for indirect IIF is an elegant approach but is limited by the availability of type VII collagen-deficient skin and the restriction to patients with reactivity against normal salt-split skin (176).

The most practical approach outside specialized laboratories to diagnose EBA is the determination of anti-type VII collagen autoantibodies by commercial ELISA or Biochip™-based indirect IFM employing the recombinant NC1 domain of type VII collagen (77, 101). Antibody levels have been shown to correlate with disease severity (177). In specialized laboratories, immunoblots and ELISA with various cell-derived and recombinant forms of type VII collagen are available (99, 178–181). Most recently, patients with predominant IgA reactivity against type VII collagen are differentiated from LAD based on the possibly more severe clinical manifestation and more refractory course compared to the anti-BP180 IgA-mediated classical LAD (115).

### Bullous systemic lupus erythematosus

BSLE is a rare AIBD in patients with SLE. Diagnostic criteria for BSLE were first proposed in 1983 by Camisa and Sharma and later revised (46, 182–184). These revised criteria include

- diagnosis of SLE based on the ACR/EULAR criteria
- vesiculobullous skin lesions predominantly in sun-exposed areas
- histopathological changes compatible with dermatitis herpetiformis
- linear or granular deposits of IgG, IgA, IgM, and complement at the BMZ by direct IFM

Lesional histopathology resembles changes observed in dermatitis herpetiformis with neutrophil-rich infiltrate in the upper dermis, dermal edema, and fibrin and neutrophils in the cavity of subepidermal blisters (185). By direct IFM, most cases reveal deposits along the BMZ of more than one Ig isotype, in many cases IgG, IgA, or IgM, whereas complement components are mostly detected in lesional skin and rarely in clinically uninvolved skin (186). In patients with linear IFM deposits, circulating autoantibodies can be often detected in indirect IFM on human salt-split skin, in most cases showing dermal binding (48). Serum autoantibodies are mainly directed against type VII collagen. In some cases, reactivity to BP180, BP230, and laminin 332 has been described (48, 51, 185, 187).

### Dermatitis herpetiformis

Diagnosis of dermatitis herpetiformis is based on the correlation of the typical clinical picture of dermatitis herpetiformis with positive direct IFM demonstrating granular deposits of IgA at the tips of dermal papillae or along the BMZ (25, 163). In the case of typical clinical manifestations but repeatedly negative direct IFM, additional minor criteria can be used to support the diagnosis. These minor criteria include the following (25):

- dermatitis herpetiformis-typical histopathological findings
- positivity of at least one of the serological tests for IgA against TG2, TG3, or endomysium
- histopathological findings in duodenal biopsy compatible with celiac disease
- HLA testing compatible with dermatitis herpetiformis/coeliac disease
- positive result of oral iodine challenge or cutaneous iodine path test
- positive therapeutic effect of a long-term gluten-free diet
- fast response to therapy with dapsone

Classical histological findings in dermatitis herpetiformis are neutrophilic microabscesses, nuclear dust, and fibrin in the papillary dermis, often accompanied by edema of dermal papillae and subepidermal split formation. Neutrophils with fibrin and polymorphonuclear cells with a variable number of eosinophils can be observed in the lumen of subepidermal blisters and the papillary dermis (140, 188). However, histological changes do not allow definite differentiation of dermatitis herpetiformis from other subepidermal AIBDs. Several serological tests are available. Anti-endomysium antibodies can be found in 60%–90% of untreated dermatitis herpetiformis patients with almost 100% specificity either using indirect IFM on monkey esophagus or using ELISA with tissue-type TG (TG2) (189, 190). IgA antibodies against epidermal TG (TG3) are a part of IgA deposits in direct IFM of dermatitis herpetiformis patients. The majority of dermatitis herpetiformis patients also have circulating anti-TG3 IgA even if anti-TG2 antibodies are absent (191–193). In the so far largest study with 242 dermatitis herpetiformis patients, 84.3% and 78.5% of sera revealed IgA reactivity against endomysium and TG2 with specificities of 100% and 99.0%, respectively (194). The European guideline of dermatitis herpetiformis recommends performing at least one serological test, i.e., either anti-endomysium IgA or anti-TG2 IgA. Serum anti-TG2 IgA is a specific marker for gluten-induced enteropathy in both dermatitis herpetiformis and coeliac disease and is recommended by the guideline. Although TG3 is regarded as an autoantigen of dermatitis herpetiformis, determination of anti-TG3 IgA is only recommended in addition to anti-TG2 IgA (25).

Genome-wide association studies (GWASs) have detected human leukocyte antigen haplotypes as major risk loci with DQ2.5 (HLA-DQA1*05 and HLA-DQB1*02), DQ2.2 (HLA-DQA1*02 and HLA-DQB1*02), DQ7.5 (HLA-DQA1*05 without HLA-DQB1*02), or DQ8 (HLA-DQB1*03:02) haplotypes detected in over 99% of individuals with celiac disease and dermatitis herpetiformis (195). However, as the frequency of these haplotypes in the population reaches 60%, their predictive value is low, but their negative predictive value is very high, as the detection of another haplotype can confidently exclude the diagnosis (196). Furthermore, challenges with either potassium iodine on healthy skin inducing dermatitis herpetiformis lesions or a gluten-free diet alleviating gastrointestinal symptoms rapidly with skin lesions improving over weeks or several months are indicative of dermatitis herpetiformis. Indirect diagnostic evidence may also come from treatment with dapsone leading to resolving symptoms after only a few days (8, 197–201).

## Future perspectives

The correct and timely diagnosis of AIBDs is paramount for the management of patients. Thus, the identification of the exact specificity of the autoantibodies including the isotype (IgG, IgA, IgM, and IgE) and target antigen is of increasing importance. Diagnosis of the exact AIBDs is relevant to initiate an appropriate therapy and to communicate the prognosis. For example, anti-p200 pemphigoid can usually be treated with lower doses of corticosteroids compared to BP, and when antibodies against laminin 332 are present in MMP, a tumor search is required. Although enormous progress has been made with respect to the development of widely available standardized assays for serum autoantibodies in AIBDs, relevant diagnostic gaps remain. As such, no test system for antibodies to the BP180 ectodomain outside the NC16A domain is available, and also test systems for the detection of IgA autoantibodies in pemphigoid diseases are missing. Implementation of the regulation (EU) 2017/746 on *in vitro* diagnostic regulation (IVDR) will be a major hurdle for both manufacturers of test systems for rare diseases, such as AIBDs, and specialized laboratories with in-house assays, also termed laboratory-developed tests. Further innovations in the field of AIBDs diagnostics can be expected regarding automation, multivariate systems, and artificial intelligence (AI)-based evaluation (202, 203).

## Author contributions

NB: Conceptualization, Project administration, Supervision, Visualization, Writing – original draft, Writing – review & editing. MH: Visualization, Writing – original draft, Writing – review & editing. IA: Writing – original draft, Writing – review & editing, Visualization. HO: Visualization, Writing – original draft, Writing – review & editing. MS: Visualization, Writing – original draft, Writing – review & editing. JP: Visualization, Writing – original draft, Writing – review & editing. AV: Visualization, Writing – original draft, Writing – review & editing. ES: Conceptualization, Funding acquisition, Supervision, Writing – original draft, Writing – review & editing.

## References

[1] Beek NV Zillikens D Schmidt E. Bullous autoimmune dermatoses-clinical features, diagnostic evaluation, and treatment options. Dtsch Arztebl Int. (2021) 118:413–20. doi: 10.3238/aerztebl.m.2021.0136 PMC8380840 34369370

[2] Kasperkiewicz M Ellebrecht CT Takahashi H Yamagami J Zillikens D Payne AS. Pemphigus. Nat Rev Dis Primers. (2017) 3:17026. doi: 10.1038/nrdp.2017.26 28492232 PMC5901732

[3] Schmidt E Kasperkiewicz M Joly P. Pemphigus. Lancet. (2019) 394:882–94. doi: 10.1016/S0140-6736(19)31778-7 31498102

[4] Stanley JR Amagai M. Pemphigus, bullous impetigo, and the staphylococcal scalded-skin syndrome. N Engl J Med. (2006) 355:1800–10. doi: 10.1056/NEJMra061111 17065642

[5] Amber KT Murrell DF Schmidt E Joly P Borradori L. Autoimmune subepidermal bullous diseases of the skin and mucosae: clinical features, diagnosis, and management. Clin Rev Allergy Immunol. (2018) 54:26–51. doi: 10.1007/s12016-017-8633-4 28779299

[6] Schmidt E Zillikens D. Pemphigoid diseases. Lancet. (2013) 381:320–32. doi: 10.1016/S0140-6736(12)61140-4 23237497

[7] Antiga E Maglie R Quintarelli L Verdelli A Bonciani D Bonciolini V. Dermatitis herpetiformis: novel perspectives. Front Immunol. (2019) 10:1290. doi: 10.3389/fimmu.2019.01290 31244841 PMC6579917

[8] Reunala T Hervonen K Salmi T. Dermatitis herpetiformis: an update on diagnosis and management. Am J Clin Dermatol. (2021) 22:329–38. doi: 10.1007/s40257-020-00584-2 PMC8068693 33432477

[9] Bertram F Brocker EB Zillikens D Schmidt E. Prospective analysis of the incidence of autoimmune bullous disorders in Lower Franconia, Germany. J Dtsch Dermatol Ges. (2009) 7:434–40. doi: 10.1111/j.1610-0387.2008.06976.x 19170813

[10] Langan SM Smeeth L Hubbard R Fleming KM Smith CJ West J. Bullous pemphigoid and pemphigus vulgaris–incidence and mortality in the UK: population based cohort study. Bmj. (2008) 337:a180. doi: 10.1136/bmj.a180 18614511 PMC2483869

[11] Marazza G Pham HC Scharer L Pedrazzetti PP Hunziker T Trueb RM. Incidence of bullous pemphigoid and pemphigus in Switzerland: a 2-year prospective study. Br J Dermatol. (2009) 161:861–8. doi: 10.1111/bjd.2009.161.issue-4 19566661

[12] Joly P Baricault S Sparsa A Bernard P Bedane C Duvert-Lehembre S. Incidence and mortality of bullous pemphigoid in France. J Invest Dermatol. (2012) 132:1998–2004. doi: 10.1038/jid.2012.35 22418872

[13] Jung M Kippes W Messer G Zillikens D Rzany B. Increased risk of bullous pemphigoid in male and very old patients: A population-based study on incidence. J Am Acad Dermatol. (1999) 41:266–8. doi: 10.1016/S0190-9622(99)70061-7 10426901

[14] Bernard P Vaillant L Labeille B Bedane C Arbeille B Denoeux JP. Incidence and distribution of subepidermal autoimmune bullous skin diseases in three French regions. Bullous Dis French Study Group Arch Dermatol. (1995) 131:48–52. doi: 10.1001/archderm.131.1.48 7826096

[15] Kridin K. Subepidermal autoimmune bullous diseases: overview, epidemiology, and associations. Immunol Res. (2018) 66:6–17. doi: 10.1007/s12026-017-8975-2 29159697

[16] Pisanti S Sharav Y Kaufman E Posner LN. Pemphigus vulgaris: incidence in Jews of different ethnic groups, according to age, sex, and initial lesion. Oral Surg Oral Med Oral Pathol. (1974) 38:382–7. doi: 10.1016/0030-4220(74)90365-X 4528670

[17] Kridin K Schmidt E. Epidemiology of pemphigus. JID Innov. (2021) 1:100004. doi: 10.1016/j.xjidi.2021.100004 34909708 PMC8659392

[18] Hubner F Konig IR Holtsche MM Zillikens D Linder R Schmidt E. Prevalence and age distribution of pemphigus and pemphigoid diseases among pediatric patients in Germany. J Eur Acad Dermatol Venereol. (2020). 10.1111/jdv.16467 32289873

[19] Borradori L Van Beek N Feliciani C Tedbirt B Antiga E Bergman R. Updated S2 K guidelines for the management of bullous pemphigoid initiated by the European Academy of Dermatology and Venereology (EADV). J Eur Acad Dermatol Venereol. (2022) 36:1689–704. doi: 10.1111/jdv.18220 35766904

[20] Joly P Horvath B Patsatsi A Uzun S Bech R Beissert S. Updated S2K guidelines on the management of pemphigus vulgaris and foliaceus initiated by the european academy of dermatology and venereology (EADV). J Eur Acad Dermatol Venereol. (2020) 34:1900–13. doi: 10.1111/jdv.16752 32830877

[21] Schmidt E Sticherling M Sardy M Eming R Goebeler M Hertl M. S2k guidelines for the treatment of pemphigus vulgaris/foliaceus and bullous pemphigoid: 2019 update. J Dtsch Dermatol Ges. (2020) 18:516–26. doi: 10.1111/ddg.14097 32413212

[22] Vorobyev A Ludwig RJ Schmidt E. Clinical features and diagnosis of epidermolysis bullosa acquisita. Expert Rev Clin Immunol. (2017) 13:157–69. doi: 10.1080/1744666X.2016.1221343 27580464

[23] Anhalt GJ. Paraneoplastic pemphigus. J Investig Dermatol Symp Proc. (2004) 9:29–33. doi: 10.1111/j.1087-0024.2004.00832.x 14870982

[24] Goletz S Probst C Komorowski L Schlumberger W Fechner K van Beek N. A sensitive and specific assay for the serological diagnosis of antilaminin 332 mucous membrane pemphigoid. Br J Dermatol. (2018). doi: 10.1111/bjd.17202 30216412

[25] Gorog A Antiga E Caproni M Cianchini G De D Dmochowski M. S2k guidelines (consensus statement) for diagnosis and therapy of dermatitis herpetiformis initiated by the European Academy of Dermatology and Venereology (EADV). J Eur Acad Dermatol Venereol. (2021) 35:1251–77. doi: 10.1111/jdv.17183 34004067

[26] Schmidt E Diaz L Amagai A Murrell D. Obituary for Professor Detlef Zillikens- creator of a powerhouse of dermatology research at the University of Lübeck, Germany. J Invest Dermatol. (2023) 143:7–8. doi: 10.1016/j.jid.2022.11.001

[27] Schmidt E Brocker EB Zillikens D. [Pemphigus. Loss of desmosomal cell-cell contact]. Hautarzt. (2000) 51:309–18. doi: 10.1007/s001050051124 10875067

[28] Kridin K Patel PM Jones VA Cordova A Amber KT. IgA pemphigus: A systematic review. J Am Acad Dermatol. (2020) 82:1386–92. doi: 10.1016/j.jaad.2019.11.059 31812619

[29] Antiga E Bech R Maglie R Genovese G Borradori L Bockle B. S2k guidelines on the management of paraneoplastic pemphigus/paraneoplastic autoimmune multiorgan syndrome initiated by the European Academy of Dermatology and Venereology (EADV). J Eur Acad Dermatol Venereol. (2023) 37(6):1118–34. doi: 10.1111/jdv.18931 PMC10806824 36965110

[30] Zimmermann J Bahmer F Rose C Zillikens D Schmidt E. Clinical and immunopathological spectrum of paraneoplastic pemphigus. J Dtsch Dermatol Ges. (2010) 8:598–606. doi: 10.1111/j.1610-0387.2010.07380_supp.x 20180886

[31] Kasperkiewicz M Zillikens D Schmidt E. Pemphigoid diseases: pathogenesis, diagnosis, and treatment. Autoimmunity. (2012) 45:55–70. doi: 10.3109/08916934.2011.606447 21923615

[32] della Torre R Combescure C Cortes B Marazza G Beltraminelli H Naldi L. Clinical presentation and diagnostic delay in bullous pemphigoid: a prospective nationwide cohort. Br J Dermatol. (2012) 167:1111–7. doi: 10.1111/bjd.2012.167.issue-5 22709136

[33] Schmidt E della Torre R Borradori L. Clinical features and practical diagnosis of bullous pemphigoid. Dermatol Clin. (2011) 29:427–38, viii-ix. doi: 10.1016/j.det.2011.03.010 21605808

[34] Stander S Schmidt E Zillikens D Ludwig RJ Kridin K. Immunological features and factors associated with mucocutaneous bullous pemphigoid - a retrospective cohort study. J Dtsch Dermatol Ges. (2021) 19(9):1289–95. doi: 10.1111/ddg.14494 34164921

[35] Huilaja L Makikallio K Tasanen K. Gestational pemphigoid. Orphanet J Rare Dis. (2014) 9:136. doi: 10.1186/s13023-014-0136-2 25178359 PMC4154519

[36] Chan LS Ahmed AR Anhalt GJ Bernauer W Cooper KD Elder MJ. The first international consensus on mucous membrane pemphigoid: definition, diagnostic criteria, pathogenic factors, medical treatment, and prognostic indicators. Arch Dermatol. (2002) 138:370–9. doi: 10.1001/archderm.138.3.370 11902988

[37] Holtsche MM Zillikens D Schmidt E. [Mucous membrane pemphigoid]. Hautarzt. (2018) 69:67–83. doi: 10.1007/s00105-017-4089-y 29242962

[38] Du G Patzelt S van Beek N Schmidt E. Mucous membrane pemphigoid. Autoimmun Rev. (2022) 21:103036. doi: 10.1016/j.autrev.2022.103036 34995762

[39] van Beek N Kridin K Buhler E Kochan AS Stander S Ludwig RJ. Evaluation of site- and autoantigen-specific characteristics of mucous membrane pemphigoid. JAMA Dermatol. (2022) 158:84–9. doi: 10.1001/jamadermatol.2021.4773 PMC8771290 34817539

[40] Kridin K van Beek N Buhler E Kochan AS Ranjbar M Beissert S. Characteristics associated with refractory course, blindness, and treatment strategy-related outcomes in patients with mucous membrane pemphigoid. JAMA Dermatol. (2023) 159:198–203. doi: 10.1001/jamadermatol.2022.5829 36630148 PMC9857489

[41] Rashid H Lamberts A Borradori L Alberti-Violetti S Barry RJ Caproni M. European guidelines (S3) on diagnosis and management of mucous membrane pemphigoid, initiated by the European Academy of Dermatology and Venereology - Part I. J Eur Acad Dermatol Venereol. (2021) 35:1750–64. doi: 10.1111/jdv.17397 PMC8457055 34245180

[42] Aromolo IF Maronese CA Moltrasio C Genovese G Marzano AV. Brunsting-Perry pemphigoid: a systematic review. Int J Dermatol. (2022) 61:1353–8. doi: 10.1111/ijd.16045 35049061

[43] Holtsche MM Goletz S Zillikens D. [Anti-p200 pemphigoid]. Hautarzt. (2019) 70:271–6. doi: 10.1007/s00105-019-4376-x 30868255

[44] Goletz S Hashimoto T Zillikens D Schmidt E. Anti-p200 pemphigoid. J Am Acad Dermatol. (2014) 71:185–91. doi: 10.1016/j.jaad.2014.02.036 24767733

[45] Kridin K Ahmed AR. Anti-p200 pemphigoid: A systematic review. Front Immunol. (2019) 10:2466. doi: 10.3389/fimmu.2019.02466 31695695 PMC6817563

[46] Maronese CA Cassano N Genovese G Foti C Vena GA Marzano AV. The intriguing links between psoriasis and bullous pemphigoid. J Clin Med. (2022) 12(1):328. doi: 10.3390/jcm12010328 36615129 PMC9821109

[47] Kasperkiewicz M Sadik CD Bieber K Ibrahim SM Manz RA Schmidt E. Epidermolysis bullosa acquisita: from pathophysiology to novel therapeutic options. J Invest Dermatol. (2016) 136:24–33. doi: 10.1038/JID.2015.356 26763420

[48] Sebaratnam DF Murrell DF. Bullous systemic lupus erythematosus. Dermatol Clin. (2011) 29:649–53. doi: 10.1016/j.det.2011.06.002 21925012

[49] Hubner F Langan EA Recke A. Lichen planus pemphigoides: from lichenoid inflammation to autoantibody-mediated blistering. Front Immunol. (2019) 10:1389. doi: 10.3389/fimmu.2019.01389 31312198 PMC6614382

[50] Yilmaz K Goletz S Pas HH van den Bos RR Blauvelt A White WL. Clinical and serological characterization of orf-induced immunobullous disease. JAMA Dermatol. (2022) 158:670–4. doi: 10.1001/jamadermatol.2022.0290 PMC8968717 35353128

[51] Schmidt E Groves R. Immunobullous disorders. In: Griffith C Barker J Bleiker T Hussain W Simpson RS, editors. Rook’s Textbook of Dermatology, part 3, chapter 50, 10th edition. Wiley-Blackwell, Chichester (2024).

[52] Yilmaz K Hammers CM Boch K Zillikens D Shimanovich I Schmidt E. Immunoglobulin M mucous membrane pemphigoid. J Dtsch Dermatol Ges. (2023) 21:285–7. doi: 10.1111/ddg.14973 36772920

[53] Rose C Brocker EB Zillikens D. Clinical, histological and immunpathological findings in 32 patients with dermatitis herpetiformis Duhring. J Dtsch Dermatol Ges. (2010) 8:265–70, -71. doi: 10.1111/j.1610-0387.2009.07292.x 19878401

[54] Buijsrogge JJ Diercks GF Pas HH Jonkman MF. The many faces of epidermolysis bullosa acquisita after serration pattern analysis by direct immunofluorescence microscopy. Br J Dermatol. (2011) 165:92–8. doi: 10.1111/bjd.2011.165.issue-1 21457208

[55] Terra JB Jonkman MF Diercks GF Pas HH. Low sensitivity of type VII collagen enzyme-linked immunosorbent assay in epidermolysis bullosa acquisita: serration pattern analysis on skin biopsy is required for diagnosis. Br J Dermatol. (2013) 169:164–7. doi: 10.1111/bjd.2013.169.issue-1 23480491

[56] Giurdanella F Diercks GF Jonkman MF Pas HH. Laboratory diagnosis of pemphigus: direct immunofluorescence remains the gold standard. Br J Dermatol. (2016) 175:185–6. doi: 10.1111/bjd.14408 26798993

[57] Meijer JM Diercks GFH de Lang EWG Pas HH Jonkman MF. Assessment of diagnostic strategy for early recognition of bullous and nonbullous variants of pemphigoid. JAMA Dermatol. (2019) 155:158–65. doi: 10.1001/jamadermatol.2018.4390 PMC6439538 30624575

[58] Sardy M Kostaki D Varga R Peris K Ruzicka T. Comparative study of direct and indirect immunofluorescence and of bullous pemphigoid 180 and 230 enzyme-linked immunosorbent assays for diagnosis of bullous pemphigoid. J Am Acad Dermatol. (2013) 69:748–53. 10.1016/j.jaad.2013.07.009 23969034

[59] Vodegel RM Jonkman MF Pas HH de Jong MC. U-serrated immunodeposition pattern differentiates type VII collagen targeting bullous diseases from other subepidermal bullous autoimmune diseases. Br J Dermatol. (2004) 151:112–8. doi: 10.1111/j.1365-2133.2004.06006.x 15270879

[60] Meijer JM Atefi I Diercks GFH Vorobyev A Zuiderveen J Meijer HJ. Serration pattern analysis for differentiating epidermolysis bullosa acquisita from other pemphigoid diseases. J Am Acad Dermatol. (2017) 78(4):754–9.e6. doi: 10.1016/j.jaad.2017.11.029 29154993

[61] Terra JB Pas HH Hertl M Dikkers FG Kamminga N Jonkman MF. Immunofluorescence serration pattern analysis as a diagnostic criterion in antilaminin-332 mucous membrane pemphigoid: immunopathological findings and clinical experience in 10 Dutch patients. Br J Dermatol. (2011) 165:815–22. doi: 10.1111/j.1365-2133.2011.10474.x 21692774

[62] Terra JB Meijer JM Jonkman MF Diercks GF. The n- vs. u-serration is a learnable criterion to differentiate pemphigoid from epidermolysis bullosa acquisita in direct immunofluorescence serration pattern analysis. Br J Dermatol. (2013) 169:100–5. doi: 10.1111/bjd.2013.169.issue-1 23489262

[63] Diercks GF Pas HH Jonkman MF. Immunofluorescence of autoimmune bullous diseases. Surg Pathol Clin. (2017) 10:505–12. doi: 10.1016/j.path.2017.01.011 28477893

[64] Holtsche MM van Beek N Kunstner A Busch H Zillikens D Schmidt E. Diagnostic value and practicability of serration pattern analysis by direct immunofluorescence microscopy in pemphigoid diseases. Acta Derm Venereol. (2021) 101:adv00410. doi: 10.2340/00015555-3748 33491096 PMC9366507

[65] Schmidt E Rashid H Marzano AV Lamberts A Di Zenzo G Diercks GFH. European Guidelines (S3) on diagnosis and management of mucous membrane pemphigoid, initiated by the European Academy of Dermatology and Venereology - Part II. J Eur Acad Dermatol Venereol. (2021) 35:1926–48. doi: 10.1111/jdv.17395 PMC8518905 34309078

[66] Shimanovich I Nitz JM Zillikens D. Multiple and repeated sampling increases the sensitivity of direct immunofluorescence testing for the diagnosis of mucous membrane pemphigoid. J Am Acad Dermatol. (2017) 77:700–5.e3. doi: 10.1016/j.jaad.2017.05.016 28676329

[67] Carey B Joshi S Abdelghani A Mee J Andiappan M Setterfield J. The optimal oral biopsy site for diagnosis of mucous membrane pemphigoid and pemphigus vulgaris. Br J Dermatol. (2020) 182:747–53. doi: 10.1111/bjd.18032 31021396

[68] Hofmann SC Gunther C Bockle BC Didona D Ehrchen J Gaskins M. S2k Guideline for the diagnosis and treatment of mucous membrane pemphigoid. J Dtsch Dermatol Ges. (2022) 20:1530–50. doi: 10.1111/ddg.14905 36354061

[69] Schmidt E Zillikens D. Modern diagnosis of autoimmune blistering skin diseases. Autoimmun Rev. (2010) 10:84–9. doi: 10.1016/j.autrev.2010.08.007 20713186

[70] Schmidt E Groves R. Immunobullous diseases. In: Griffith C Barker J Chalmers Bleiker T Creamer D, editors. Rook’s Textbook of Dermatology, part 3, chapter 50, 9th edition. Wiley-Blackwell, Chichester (2016). p. 1–56.

[71] Gao Y Qian H Hashimoto T Li X. Potential contribution of anti-p200 autoantibodies to mucosal lesions in anti-p200 pemphigoid. Front Immunol. (2023) 14:1118846. doi: 10.3389/fimmu.2023.1118846 36761755 PMC9905711

[72] Kelly SE Wojnarowska F. The use of chemically split tissue in the detection of circulating anti-basement membrane zone antibodies in bullous pemphigoid and cicatricial pemphigoid. Br J Dermatol. (1988) 118:31–40. doi: 10.1111/j.1365-2133.1988.tb01747.x 3277659

[73] Gammon WR Briggaman RA Inman AO Queen LL 3rd Wheeler CE. Differentiating anti-lamina lucida and anti-sublamina densa anti-BMZ antibodies by indirect immunofluorescence on 1.0 M sodium chloride-separated skin. J Invest Dermatol. (1984) 82:139–44. doi: 10.1111/1523-1747.ep12259692 6363567

[74] Shornick JK Bangert JL Freeman RG Gilliam JN. Herpes gestationis: clinical and histologic features of twenty-eight cases. J Am Acad Dermatol. (1983) 8:214–24. doi: 10.1016/S0190-9622(83)70027-7 6338065

[75] Chimanovitch I Schmidt E Messer G Dopp R Partscht K Brocker EB. IgG1 and IgG3 are the major immunoglobulin subclasses targeting epitopes within the NC16A domain of BP180 in pemphigoid gestationis. J Invest Dermatol. (1999) 113:140–2. doi: 10.1046/j.1523-1747.1999.00622.x 10417635

[76] Blocker IM Dahnrich C Probst C Komorowski L Saschenbrecker S Schlumberger W. Epitope mapping of BP230 leading to a novel enzyme-linked immunosorbent assay for autoantibodies in bullous pemphigoid. Br J Dermatol. (2012) 166:964–70. 10.1111/j.1365-2133.2012.10820.x 22242606

[77] Holtsche MM van Beek N Hashimoto T Di Zenzo G Zillikens D Prost-Squarcioni C. Diagnosis of epidermolysis bullosa acquisita: multicentre comparison of different assays for serum anti-type VII collagen reactivity. Acta Derm Venereol. (2021) 101:adv00420. doi: 10.2340/00015555-3774 33686442 PMC9366678

[78] Ishii K Amagai M Hall RP Hashimoto T Takayanagi A Gamou S. Characterization of autoantibodies in pemphigus using antigen-specific enzyme-linked immunosorbent assays with baculovirus-expressed recombinant desmogleins. J Immunol. (1997) 159:2010–7. doi: 10.4049/jimmunol.159.4.2010 9257868

[79] Schmidt E Dahnrich C Rosemann A Probst C Komorowski L Saschenbrecker S. Novel ELISA systems for antibodies to desmoglein 1 and 3: correlation of disease activity with serum autoantibody levels in individual pemphigus patients. Exp Dermatol. (2010) 19:458–63. doi: 10.1111/j.1600-0625.2010.01069.x 20163452

[80] Sitaru C Dahnrich C Probst C Komorowski L Blocker I Schmidt E. Enzyme-linked immunosorbent assay using multimers of the 16th non-collagenous domain of the BP180 antigen for sensitive and specific detection of pemphigoid autoantibodies. Exp Dermatol. (2007) 16:770–7. doi: 10.1111/j.1600-0625.2007.00592.x 17697150

[81] van Beek N Dahnrich C Johannsen N Lemcke S Goletz S Hubner F. Prospective studies on the routine use of a novel multivariant enzyme-linked immunosorbent assay for the diagnosis of autoimmune bullous diseases. J Am Acad Dermatol. (2017) 76:889–94.e5. doi: 10.1016/j.jaad.2016.11.002 28038887

[82] Kobayashi M Amagai M Kuroda-Kinoshita K Hashimoto T Shirakata Y Hashimoto K. BP180 ELISA using bacterial recombinant NC16a protein as a diagnostic and monitoring tool for bullous pemphigoid. J Dermatol Sci. (2002) 30:224–32. doi: 10.1016/S0923-1811(02)00109-3 12443845

[83] Probst C Schlumberger W Stocker W Recke A Schmidt E Hashimoto T. Development of ELISA for the specific determination of autoantibodies against envoplakin and periplakin in paraneoplastic pemphigus. Clin Chim Acta. (2009) 410:13–8. doi: 10.1016/j.cca.2009.08.022 19737550

[84] Harman KE Seed PT Gratian MJ Bhogal BS Challacombe SJ Black MM. The severity of cutaneous and oral pemphigus is related to desmoglein 1 and 3 antibody levels. Br J Dermatol. (2001) 144:775–80. doi: 10.1046/j.1365-2133.2001.04132.x 11298536

[85] Schmidt E Obe K Brocker EB Zillikens D. Serum levels of autoantibodies to BP180 correlate with disease activity in patients with bullous pemphigoid. Arch Dermatol. (2000) 136:174–8. doi: 10.1001/archderm.136.2.174 10677092

[86] Kim JH Kim YH Kim S Noh EB Kim SE Vorobyev A. Serum levels of anti-type VII collagen antibodies detected by enzyme-linked immunosorbent assay in patients with epidermolysis bullosa acquisita are correlated with the severity of skin lesions. J Eur Acad Dermatol Venereol. (2012) 27:e224–30. 10.1111/j.1468-3083.2012.04617.x 22731917

[87] Murrell DF Pena S Joly P Marinovic B Hashimoto T Diaz LA. Diagnosis and management of pemphigus: Recommendations of an international panel of experts. J Am Acad Dermatol. (2020) 82:575–85.e1. doi: 10.1016/j.jaad.2018.02.021 29438767 PMC7313440

[88] Kasperkiewicz M Dahnrich C Probst C Komorowski L Stocker W Schlumberger W. Novel assay for detecting celiac disease-associated autoantibodies in dermatitis herpetiformis using deamidated gliadin-analogous fusion peptides. J Am Acad Dermatol. (2012) 66:583–8. doi: 10.1016/j.jaad.2011.02.025 21840083

[89] van Beek N Rentzsch K Probst C Komorowski L Kasperkiewicz M Fechner K. Serological diagnosis of autoimmune bullous skin diseases: Prospective comparison of the BIOCHIP mosaic-based indirect immunofluorescence technique with the conventional multi-step single test strategy. Orphanet J Rare Dis. (2012) 7:49. doi: 10.1186/1750-1172-7-49 22876746 PMC3533694

[90] Ozkesici B Mutlu D Donmez L Uzun S. The value of the BIOCHIP mosaic-based indirect immunofluorescence technique in the diagnosis of pemphigus and bullous pemphigoid in turkish patients. Acta Dermatovenerol Croat. (2017) 25:202–9. 29252172

[91] Russo I Saponeri A Peserico A Alaibac M. The use of biochip immunofluorescence microscopy for the diagnosis of Pemphigus vulgaris. Acta Histochem. (2014) 116:713–6. doi: 10.1016/j.acthis.2013.12.012 24485334

[92] Sadik CD Pas HH Bohlmann MK Mousavi S Benoit S Sardy M. Value of BIOCHIP technology in the serological diagnosis of pemphigoid gestationis. Acta Derm Venereol. (2017) 97:128–30. doi: 10.2340/00015555-2460 27174635

[93] Tampoia M Zucano A Villalta D Antico A Bizzaro N. Anti-skin specific autoantibodies detected by a new immunofluorescence multiplex biochip method in patients with autoimmune bullous diseases. Dermatology. (2012) 225:37–44. doi: 10.1159/000339776 22907099

[94] Xuan RR Yang A Murrell DF. New biochip immunofluorescence test for the serological diagnosis of pemphigus vulgaris and foliaceus: A review of the literature. Int J Womens Dermatol. (2018) 4:102–8. doi: 10.1016/j.ijwd.2017.10.001 PMC5986232 29872685

[95] Yang A Xuan R Melbourne W Tran K Murrell DF. Validation of the BIOCHIP test for the diagnosis of bullous pemphigoid, pemphigus vulgaris and pemphigus foliaceous. J Eur Acad Dermatol Venereol. (2020) 34(1):153–60. doi: 10.1111/jdv.15770 31260565

[96] Zarian H Saponeri A Michelotto A Zattra E Belloni-Fortina A Alaibac M. Biochip technology for the serological diagnosis of bullous pemphigoid. ISRN Dermatol. (2012) 2012:237802. doi: 10.5402/2012/237802 23346412 PMC3533605

[97] Hocke J Krauth J Krause C Gerlach S Warnemunde N Affeldt K. Computer-aided classification of indirect immunofluorescence patterns on esophagus and split skin for the detection of autoimmune dermatoses. Front Immunol. (2023) 14:1111172. doi: 10.3389/fimmu.2023.1111172 36926325 PMC10013071

[98] Marzano AV Cozzani E Biasin M Russo I Alaibac M. The use of Biochip immunofluorescence microscopy for the serological diagnosis of epidermolysis bullosa acquisita. Arch Dermatol Res. (2016) 308:273–6. doi: 10.1007/s00403-016-1632-0 26895535

[99] Seta V Aucouturier F Bonnefoy J Le Roux-Villet C Pendaries V Alexandre M. Comparison of 3 type VII collagen (C7) assays for serologic diagnosis of epidermolysis bullosa acquisita (EBA). J Am Acad Dermatol. (2016) 74:1166–72. doi: 10.1016/j.jaad.2016.01.005 26947449

[100] Mindorf S Dettmann IM Kruger S Fuhrmann T Rentzsch K Karl I. Routine detection of serum antidesmocollin autoantibodies is only useful in patients with atypical pemphigus. Exp Dermatol. (2017) 26:1267–70. doi: 10.1111/exd.13409 28815795

[101] Komorowski L Muller R Vorobyev A Probst C Recke A Jonkman MF. Sensitive and specific assays for routine serological diagnosis of epidermolysis bullosa acquisita. J Am Acad Dermatol. (2013) 68:e89–95. doi: 10.1016/j.jaad.2011.12.032 22341608

[102] Goletz S Giurdanella F Holtsche MM Nijenhuis M Horvath B Diercks GFH. Comparison of two diagnostic assays for anti-laminin 332 mucous membrane pemphigoid. Front Immunol. (2021) 12:773720. doi: 10.3389/fimmu.2021.773720 34899726 PMC8657402

[103] van Beek N Kruger S Fuhrmann T Lemcke S Goletz S Probst C. Multicenter prospective study on multivariant diagnostics of autoimmune bullous dermatoses using the BIOCHIP technology. J Am Acad Dermatol. (2020) 83:1315–22. doi: 10.1016/j.jaad.2020.01.049 32004645

[104] Commin MH Schmidt E Duvert-Lehembre S Lasek A Morice C Estival JL. Clinical and immunological features and outcome of anti-p200 pemphigoid. Br J Dermatol. (2016) 175:776–81. doi: 10.1111/bjd.2016.175.issue-4 27037896

[105] Groth S Recke A Vafia K Ludwig RJ Hashimoto T Zillikens D. Development of a simple enzyme-linked immunosorbent assay for the detection of autoantibodies in anti-p200 pemphigoid. Br J Dermatol. (2011) 164:76–82. doi: 10.1111/bjd.2010.164.issue-1 20854435

[106] Zillikens D Kawahara Y Ishiko A Shimizu H Mayer J Rank CV. A novel subepidermal blistering disease with autoantibodies to a 200-kDa antigen of the basement membrane zone. J Invest Dermatol. (1996) 106:1333–8. doi: 10.1111/1523-1747.ep12349283 8752680

[107] Dainichi T Kurono S Ohyama B Ishii N Sanzen N Hayashi M. Anti-laminin gamma-1 pemphigoid. Proc Natl Acad Sci U S A. (2009) 106:2800–5. doi: 10.1073/pnas.0809230106 PMC2650346 19196964

[108] Liu W Sun X Gao Y Li H Shi L Cheng L. A Chinese case of concurrent anti-laminin gamma1 pemphigoid and anti-laminin 332-type mucous membrane pemphigoid. J Dermatol. (2023) 50:e69–71. doi: 10.1111/1346-8138.16513 35811504

[109] Kuang W Qian H Zhang Q Li W Hashimoto T Zeng X. Case Report: Mucous Membrane Pemphigoid With IgG and IgA Anti-Laminin gamma1 Antibodies and IgA Anti-Laminin alpha5 Antibodies. Front Immunol. (2022) 13:903174. doi: 10.3389/fimmu.2022.903174 35720393 PMC9198329

[110] Li X Qian H Ishii N Yamaya M Fukuda H Mukai H. A case of concurrent antilaminin gamma1 pemphigoid and antilaminin-332-type mucous membrane pemphigoid. Br J Dermatol. (2014) 171:1257–9. doi: 10.1111/bjd.13107 25262782

[111] Lazarova Z Sitaru C Zillikens D Yancey KB. Comparative analysis of methods for detection of anti-laminin 5 autoantibodies in patients with anti-epiligrin cicatricial pemphigoid. J Am Acad Dermatol. (2004) 51:886–92. doi: 10.1016/j.jaad.2004.06.004 15583578

[112] Marinkovich MP Taylor TB Keene DR Burgeson RE Zone JJ. LAD-1, the linear IgA bullous dermatosis autoantigen, is a novel 120-kDa anchoring filament protein synthesized by epidermal cells. J Invest Dermatol. (1996) 106:734–8. doi: 10.1111/1523-1747.ep12345782 8618013

[113] Schmidt E Skrobek C Kromminga A Hashimoto T Messer G Brocker EB. Cicatricial pemphigoid: IgA and IgG autoantibodies target epitopes on both intra- and extracellular domains of bullous pemphigoid antigen 180. Br J Dermatol. (2001) 145:778–83. doi: 10.1046/j.1365-2133.2001.04471.x 11736901

[114] Opelka B Schmidt E Goletz S. Type XVII collagen: Relevance of distinct epitopes, complement-independent effects, and association with neurological disorders in pemphigoid disorders. Front Immunol. (2022) 13:948108. doi: 10.3389/fimmu.2022.948108 36032160 PMC9400597

[115] Becker M Schumacher N Schmidt E Zillikens D Sadik CD. Evaluation and comparison of clinical and laboratory characterstics of patients with IgA epidermolysis bullosa acquisita, linear IgA bullous dermatosis, and IgG epidermolysis bullosa acquisita. JAMA Dermatol. (2021) 157(8):917–23. doi: 10.1001/jamadermatol.2021.0762 PMC8223139 34160564

[116] Zillikens D Herzele K Georgi M Schmidt E Chimanovitch I Schumann H. Autoantibodies in a subgroup of patients with linear IgA disease react with the NC16A domain of BP1801. J Invest Dermatol. (1999) 113:947–53. doi: 10.1046/j.1523-1747.1999.00808.x 10594735

[117] Csorba K Schmidt S Florea F Ishii N Hashimoto T Hertl M. Development of an ELISA for sensitive and specific detection of IgA autoantibodies against BP180 in pemphigoid diseases. Orphanet J Rare Dis. (2011) 6:31. doi: 10.1186/1750-1172-6-31 21619684 PMC3126693

[118] Bernard P Antonicelli F Bedane C Joly P Le Roux-Villet C Duvert-Lehembre S. Prevalence and clinical significance of anti-laminin 332 autoantibodies detected by a novel enzyme-linked immunosorbent assay in mucous membrane pemphigoid. JAMA Dermatol. (2013) 149:533–40. doi: 10.1001/jamadermatol.2013.1434 23426192

[119] Chiorean R Danescu S Virtic O Mustafa MB Baican A Lischka A. Molecular diagnosis of anti-laminin 332 (epiligrin) mucous membrane pemphigoid. Orphanet J Rare Dis. (2018) 13:111. doi: 10.1186/s13023-018-0855-x 29980216 PMC6035451

[120] Domloge-Hultsch N Gammon WR Briggaman RA Gil SG Carter WG Yancey KB. Epiligrin, the major human keratinocyte integrin ligand, is a target in both an acquired autoimmune and an inherited subepidermal blistering skin disease. J Clin Invest. (1992) 90:1628–33. doi: 10.1172/JCI116033 PMC443212 1401088

[121] Giurdanella F Nijenhuis AM Diercks GFH Jonkman MF Pas HH. Keratinocyte footprint assay discriminates antilaminin-332 pemphigoid from all other forms of pemphigoid diseases. Br J Dermatol. (2020) 182:373–81. doi: 10.1111/bjd.18129 PMC7027452 31090065

[122] Schepens I Jaunin F Begre N Laderach U Marcus K Hashimoto T. The protease inhibitor alpha-2-macroglobulin-like-1 is the p170 antigen recognized by paraneoplastic pemphigus autoantibodies in human. PloS One. (2010) 5:e12250. doi: 10.1371/journal.pone.0012250 20805888 PMC2923615

[123] Tsuchisaka A Numata S Teye K Natsuaki Y Kawakami T Takeda Y. Epiplakin is a paraneoplastic pemphigus autoantigen and related to bronchiolitis obliterans in Japanese patients. J Invest Dermatol. (2016) 136:399–408. doi: 10.1038/JID.2015.408 26802236

[124] Anhalt GJ. Paraneoplastic pemphigus. Adv Dermatol. (1997) 12:77–96. 8973736

[125] Ohzono A Sogame R Li X Teye K Tsuchisaka A Numata S. Clinical and immunological findings in 104 cases of paraneoplastic pemphigus. Br J Dermatol. (2015) 173:1447–52. doi: 10.1111/bjd.14162 26358412

[126] Hashimoto T Qian H Ishii N Nakama T Tateishi C Tsuruta D. Classification and antigen molecules of autoimmune bullous diseases. Biomolecules. (2023) 13. doi: 10.3390/biom13040703 PMC10135556 37189450

[127] Li X Qian H Sogame R Hirako Y Tsuruta D Ishii N. Integrin beta4 is a major target antigen in pure ocular mucous membrane pemphigoid. Eur J Dermatol. (2016) 26:247–53. 10.1684/ejd.2016.2772 27193492

[128] Maglie R De Almeida CV Baffa ME Bianchi B Caproni M Di Zenzo G. Anti-beta4 integrin autoantibodies in patients with mucous membrane pemphigoid: A retrospective analysis from a tertiary centre in Italy. J Eur Acad Dermatol Venereol. (2023) 37(2):e249–51. doi: 10.1111/jdv.18617 36166640

[129] van Beek N Luttmann N Huebner F Recke A Karl I Schulze FS. Correlation of serum levels of IgE autoantibodies against BP180 with bullous pemphigoid disease activity. JAMA Dermatol. (2017) 153:30–8. doi: 10.1001/jamadermatol.2016.3357 27829102

[130] Messingham KA Noe MH Chapman MA Giudice GJ Fairley JA. A novel ELISA reveals high frequencies of BP180-specific IgE production in bullous pemphigoid. J Immunol Methods. (2009) 346:18–25. doi: 10.1016/j.jim.2009.04.013 19422829 PMC2703696

[131] Yasukochi A Teye K Ishii N Hashimoto T. Clinical and immunological studies of 332 Japanese patients tentatively diagnosed as anti-BP180-type mucous membrane pemphigoid: A novel BP180 C-terminal domain enzyme-linked immunosorbent assay. Acta Derm Venereol. (2016) 96:762–7. 10.2340/00015555-2407 26984589

[132] Hashimoto T Ohzono A Teye K Numata S Hiroyasu S Tsuruta D. Detection of IgE autoantibodies to BP180 and BP230 and their relationship to clinical features in bullous pemphigoid. Br J Dermatol. (2017) 177:141–51. doi: 10.1111/bjd.15114 27716903

[133] van Beek N Schulze FS Zillikens D Schmidt E. IgE-mediated mechanisms in bullous pemphigoid and other autoimmune bullous diseases. Expert Rev Clin Immunol. (2016) 12:267–77. doi: 10.1586/1744666X.2016.1123092 26588556

[134] Ali S Kelly C Challacombe SJ Donaldson AN Bhogal BS Setterfield JF. Serum and salivary IgG and IgA antibodies to desmoglein 3 in mucosal pemphigus vulgaris. Br J Dermatol. (2016) 175:113–21. doi: 10.1111/bjd.14410 26799252

[135] Ali S Kelly C Challacombe SJ Donaldson AN Dart JK Gleeson M. Salivary IgA and IgG antibodies to bullous pemphigoid 180 noncollagenous domain 16a as diagnostic biomarkers in mucous membrane pemphigoid. Br J Dermatol. (2016) 174:1022–9. doi: 10.1111/bjd.14351 26676445

[136] Lever WF. Pemphigus. Med (Baltimore). (1953) 32:1–123. doi: 10.1097/00005792-195302000-00001 13024494

[137] van Beek N Schumacher N Rose C Schmidt E Zillikens D. [Modern diagnostics of autoimmune bullous diseases]. Pathologe. (2020) 41:317–25. doi: 10.1007/s00292-020-00795-8 32542511

[138] Stander S Hammers CM Vorobyev A Schmidt E Zillikens D Ghorbanalipoor S. The impact of lesional inflammatory cellular infiltrate on the phenotype of bullous pemphigoid. J Eur Acad Dermatol Venereol. (2021) 35:1702–11. doi: 10.1111/jdv.17303 33896060

[139] Vale E Dimatos OC Porro AM Santi CG. Consensus on the treatment of autoimmune bullous dermatoses: dermatitis herpetiformis and linear IgA bullous dermatosis - Brazilian Society of Dermatology. Bras Dermatol. (2019) 94:48–55. doi: 10.1590/abd1806-4841.2019940208 PMC6544034 31166403

[140] Imber MJ Kibbi AG Mihm MC Jr. Dermatitis herpetiformis: histopathologic findings Clin Dermatol. (1991) 9:289–93. doi: 10.1016/0738-081X(91)90020-L 1806216

[141] Rose C Schmidt E Kerstan A Thoma-Uszynski S Wesselmann U Kasbohrer U. Histopathology of anti-laminin 5 mucous membrane pemphigoid. J Am Acad Dermatol. (2009) 61:433–40. doi: 10.1016/j.jaad.2009.02.012 19700013

[142] Rose C Weyers W Denisjuk N Hillen U Zillikens D Shimanovich I. Histopathology of anti-p200 pemphigoid. Am J Dermatopathol. (2007) 29:119–24. doi: 10.1097/DAD.0b013e31803326e6 17414431

[143] Glauser S Rutz M Cazzaniga S Hegyi I Borradori L Beltraminelli H. Diagnostic value of immunohistochemistry on formalin-fixed, paraffin-embedded skin biopsy specimens for bullous pemphigoid. Br J Dermatol. (2016) 175:988–93. doi: 10.1111/bjd.2016.175.issue-5 27105821

[144] Pfaltz K Mertz K Rose C Scheidegger P Pfaltz M Kempf W. C3d immunohistochemistry on formalin-fixed tissue is a valuable tool in the diagnosis of bullous pemphigoid of the skin. J Cutan Pathol. (2010) 37:654–8. doi: 10.1111/j.1600-0560.2009.01450.x 19863700

[145] Shimanovich I Nitz JM Witte M Zillikens D Rose C. Immunohistochemical diagnosis of mucous membrane pemphigoid. J Oral Pathol Med. (2018) 47:613–9. doi: 10.1111/jop.12732 29752861

[146] Villani AP Chouvet B Kanitakis J. Application of C4d immunohistochemistry on routinely processed tissue sections for the diagnosis of autoimmune bullous dermatoses. Am J Dermatopathol. (2016) 38:186–8. doi: 10.1097/DAD.0000000000000333 25793311

[147] Kasperkiewicz M Lai O Kim G DeClerck B Woodley DT Zillikens D. Immunoglobulin and complement immunohistochemistry on paraffin sections in autoimmune bullous diseases: A systematic review and meta-analysis. Am J Dermatopathol. (2021) 43:689–99. doi: 10.1097/DAD.0000000000001817 33055534

[148] Schmidt E Goebeler M Hertl M Sardy M Sitaru C Eming R. S2k guideline for the diagnosis of pemphigus vulgaris/foliaceus and bullous pemphigoid. J Dtsch Dermatol Ges. (2015) 13:713–27. doi: 10.1111/ddg.12612 26110729

[149] van Beek N Kruger S Fuhrmann T Lemcke S Goletz S Probst C. Multicenter prospective study on multivariant diagnostics of autoimmune bullous dermatoses using the BIOCHIP(TM) technology. J Am Acad Dermatol. (2020) 83(5):1315–22. doi: 10.1016/j.jaad.2020.01.049 32004645

[150] Hashimoto T Nishikawa T. Nomenclature for diseases with IgA antikeratinocyte cell surface autoantibodies. Br J Dermatol. (2015) 173:868–9. doi: 10.1111/bjd.13813 25823861

[151] Tsuruta D Ishii N Hamada T Ohyama B Fukuda S Koga H. IgA pemphigus. Clin Dermatol. (2011) 29:437–42. doi: 10.1016/j.clindermatol.2011.01.014 21679872

[152] Boch K Hammers CM Goletz S Kamaguchi M Ludwig RJ Schneider SW. Immunoglobulin M pemphigoid. J Am Acad Dermatol. (2021) 85:1486–92. doi: 10.1016/j.jaad.2021.01.017 33453342

[153] Rashid H Meijer JM Diercks GFH Sieben NE Bolling MC Pas HH. Assessment of diagnostic strategy for mucous membrane pemphigoid. JAMA Dermatol. (2021) 157:780–7. doi: 10.1001/jamadermatol.2021.1036 PMC8082433 33909024

[154] Murakami H Nishioka S Setterfield J Bhogal BS Black MM Zillikens D. Analysis of antigens targeted by circulating IgG and IgA autoantibodies in 50 patients with cicatricial pemphigoid. J Dermatol Sci. (1998) 17:39–44. doi: 10.1016/S0923-1811(97)00067-4 9651827

[155] Shimanovich I Brocker EB Zillikens D. Pemphigoid gestationis: new insights into the pathogenesis lead to novel diagnostic tools. BJOG. (2002) 109:970–6. doi: 10.1111/j.1471-0528.2002.01016.x 12269691

[156] Sadik CD Lima AL Zillikens D. Pemphigoid gestationis: Toward a better understanding of the etiopathogenesis. Clin Dermatol. (2016) 34:378–82. doi: 10.1016/j.clindermatol.2016.02.010 27265076

[157] Powell AM Sakuma-Oyama Y Oyama N Albert S Bhogal B Kaneko F. Usefulness of BP180 NC16a enzyme-linked immunosorbent assay in the serodiagnosis of pemphigoid gestationis and in differentiating between pemphigoid gestationis and pruritic urticarial papules and plaques of pregnancy. Arch Dermatol. (2005) 141:705–10. doi: 10.1001/archderm.141.6.705 15967916

[158] Sitaru C Powell J Messer G Brocker EB Wojnarowska F Zillikens D. Immunoblotting and enzyme-linked immunosorbent assay for the diagnosis of pemphigoid gestationis. Obstet Gynecol. (2004) 103:757–63. doi: 10.1097/01.AOG.0000115506.76104.ad 15051570

[159] Barnadas MA Rubiales MV Gonzalez MJ Puig L Garcia P Baselga E. Enzyme-linked immunosorbent assay (ELISA) and indirect immunofluorescence testing in a bullous pemphigoid and pemphigoid gestationis. Int J Dermatol. (2008) 47:1245–9. doi: 10.1111/j.1365-4632.2008.03824.x 19126009

[160] Wojnarowska F Marsden RA Bhogal B Black MM. Chronic bullous disease of childhood, childhood cicatricial pemphigoid, and linear IgA disease of adults. A comparative study demonstrating clinical and immunopathologic overlap. J Am Acad Dermatol. (1988) 19:792–805. doi: 10.1016/S0190-9622(88)70236-4 3056993

[161] Juratli HA Sardy M. [Linear IgA bullous dermatosis]. Hautarzt. (2019) 70:254–9. doi: 10.1007/s00105-019-4377-9 30874843

[162] Becker M Schumacher N Schmidt E Zillikens D Sadik CD. Evaluation and comparison of clinical and iLaboratory characteristics of patients with IgA epidermolysis bullosa acquisita, linear IgA bullous dermatosis, and IgG epidermolysis bullosa acquisita. JAMA Dermatol. (2021) 157:917–23. doi: 10.1001/jamadermatol.2021.0762 PMC8223139 34160564

[163] Chorzelski TP Beutner EH Jablonska S Blaszczyk M Triftshauser C. Immunofluorescence studies in the diagnosis of dermatitis herpetiformis and its differentiation from bullous pemphigoid. J Invest Dermatol. (1971) 56:373–80. doi: 10.1111/1523-1747.ep12261260 4104140

[164] Meijer JM Diercks GF Schmidt E Pas HH Jonkman MF. Laboratory diagnosis and clinical profile of anti-p200 pemphigoid. JAMA Dermatol. (2016) 152:897–904. doi: 10.1001/jamadermatol.2016.1099 27167149

[165] Dainichi T Koga H Tsuji T Ishii N Ohyama B Ueda A. From anti-p200 pemphigoid to anti-laminin gamma1 pemphigoid. J Dermatol. (2010) 37:231–8. doi: 10.1111/j.1346-8138.2009.00793.x 20507386

[166] Koga H Ishii N Dainichi T Tsuruta D Hamada T Ohata C. An attempt to develop mouse model for anti-laminin gamma1 pemphigoid. J Dermatol Sci. (2013) 70:108–15. doi: 10.1016/j.jdermsci.2013.01.001 23410740

[167] Vafia K Groth S Beckmann T Hirose M Dworschak J Recke A. Pathogenicity of autoantibodies in anti-p200 pemphigoid. PloS One. (2012) 7:e41769. doi: 10.1371/journal.pone.0041769 22911854 PMC3404064

[168] Goletz S Pigors M Lari TR Hammers CM Wang Y Emtenani S. Laminin beta4 is a constituent of the cutaneous basement membrane zone and additional autoantigen of anti-p200 pemphigoid. J Am Acad Dermatol. (2024) 90(4):790–7. doi: 10.1016/j.jaad.2023.11.014 37992812

[169] Goletz S Probst C Komorowski L Radzimski C Mindorf S Holtsche MM. Sensitive and specific assay for the serological diagnosis of anti-p200 pemphigoid based on recombinant laminin β4. Br J Dermatol. (2024) 28:ljae136. doi: 10.1093/bjd/ljae136 38544457

[170] Prost-Squarcioni C Caux F Schmidt E Jonkman MF Vassileva S Kim SC. International Bullous Diseases Group: consensus on diagnostic criteria for epidermolysis bullosa acquisita. Br J Dermatol. (2018) 179:30–41. doi: 10.1111/bjd.16138 29165796

[171] Prost C Labeille B Chaussade V Guillaume JC Martin N Dubertret L. Immunoelectron microscopy in subepidermal autoimmune bullous diseases: a prospective study of IgG and C3 bound in *vivo* in 32 patients. J Invest Dermatol. (1987) 89:567–73. doi: 10.1111/1523-1747.ep12461226 3316411

[172] Nieboer C Boorsma DM Woerdeman MJ Kalsbeek GL. Epidermolysis bullosa acquisita. Immunofluorescence, electron microscopic and immunoelectron microscopic studies in four patients. Br J Dermatol. (1980) 102:383–92. doi: 10.1111/j.1365-2133.1980.tb06550.x 6992836

[173] De Jong MC Bruins S Heeres K Jonkman MF Nieboer C Boorsma DM. Bullous pemphigoid and epidermolysis bullosa acquisita. Differentiation by fluorescence overlay antigen mapping. Arch Dermatol. (1996) 132:151–7. doi: 10.1001/archderm.132.2.151 8629822

[174] Wozniak K Kazama T Kowalewski C. A practical technique for differentiation of subepidermal bullous diseases: localization of in *vivo*-bound IgG by laser scanning confocal microscopy. Arch Dermatol. (2003) 139:1007–11. doi: 10.1001/archderm.139.8.1007 12925388

[175] Witte M Zillikens D Schmidt E. Diagnosis of autoimmune blistering diseases. Front Med (Lausanne). (2018) 5:296. doi: 10.3389/fmed.2018.00296 30450358 PMC6224342

[176] Vodegel RM Kiss M Cjm De Jong M Pas HH Altmayer A Molnar K. The use of skin substrates deficient in basement membrane molecules for the diagnosis of subepidermal autoimmune bullous disease. Eur J Dermatol. (1998) 8:83–5. doi: 10.1016/S0923-1811(98)83248-9 9649654

[177] Kim JH Kim YH Kim S Noh EB Kim SE Vorobyev A. Serum levels of anti-type VII collagen antibodies detected by enzyme-linked immunosorbent assay in patients with epidermolysis bullosa acquisita are correlated with the severity of skin lesions. J Eur Acad Dermatol Venereol. (2013) 27:e224–30. doi: 10.1111/j.1468-3083.2012.04617.x 22731917

[178] Chen M Chan LS Cai X O’Toole EA Sample JC Woodley DT. Development of an ELISA for rapid detection of anti-type VII collagen autoantibodies in epidermolysis bullosa acquisita. J Invest Dermatol. (1997) 108:68–72. doi: 10.1111/1523-1747.ep12285634 8980290

[179] Calabresi V Sinistro A Cozzani E Cerasaro C Lolicato F Muscianese M. Sensitivity of different assays for the serological diagnosis of epidermolysis bullosa acquisita: analysis of a cohort of 24 Italian patients. J Eur Acad Dermatol Venereol. (2014) 28:483–90. doi: 10.1111/jdv.12129 24321031

[180] Hashimoto T Jin Z Ishii N. Clinical and immunological studies for 105 Japanese seropositive patients of epidermolysis bullosa acquisita examined at Kurume University. Expert Rev Clin Immunol. (2016) 12:895–902. doi: 10.1080/1744666X.2016.1196136 27247994

[181] Tanaka H Ishida-Yamamoto A Hashimoto T Hiramoto K Harada T Kawachi Y. A novel variant of acquired epidermolysis bullosa with autoantibodies against the central triple-helical domain of type VII collagen. Lab Invest. (1997) 77:623–32. 9426400

[182] Odonwodo A Vashisht P. Bullous Systemic Lupus Erythematosus. Treasure Island (FL: StatPearls (2023). 32491377

[183] Camisa C Sharma HM. Vesiculobullous systemic lupus erythematosus. Report of two cases and a review of the literature. J Am Acad Dermatol. (1983) 9:924–33. doi: 10.1016/S0190-9622(83)70210-0 6358284

[184] Camisa C Grimwood RE. Indirect immunofluorescence in vesiculobullous eruption of systemic lupus erythematosus. J Invest Dermatol. (1986) 86:606. doi: 10.1111/1523-1747.ep12355583 3528314

[185] Gammon WR Briggaman RA. Bullous SLE: a phenotypically distinctive but immunologically heterogeneous bullous disorder. J Invest Dermatol. (1993) 100:28S–34S. doi: 10.1111/1523-1747.ep12355210 8423389

[186] Vassileva S. Bullous systemic lupus erythematosus. Clin Dermatol. (2004) 22:129–38. doi: 10.1016/j.clindermatol.2003.12.020 15234014

[187] Chan LS Lapiere JC Chen M Traczyk T Mancini AJ Paller AS. Bullous systemic lupus erythematosus with autoantibodies recognizing multiple skin basement membrane components, bullous pemphigoid antigen 1, laminin-5, laminin-6, and type VII collagen. Arch Dermatol. (1999) 135:569–73. doi: 10.1001/archderm.135.5.569 10328198

[188] Warren SJ Cockerell CJ. Characterization of a subgroup of patients with dermatitis herpetiformis with nonclassical histologic features. Am J Dermatopathol. (2002) 24:305–8. doi: 10.1097/00000372-200208000-00003 12142608

[189] Dahlbom I Korponay-Szabo IR Kovacs JB Szalai Z Maki M Hansson T. Prediction of clinical and mucosal severity of coeliac disease and dermatitis herpetiformis by quantification of IgA/IgG serum antibodies to tissue transglutaminase. J Pediatr Gastroenterol Nutr. (2010) 50:140–6. doi: 10.1097/MPG.0b013e3181a81384 19841593

[190] Kumar V Jarzabek-Chorzelska M Sulej J Rajadhyaksha M Jablonska S. Tissue transglutaminase and endomysial antibodies-diagnostic markers of gluten-sensitive enteropathy in dermatitis herpetiformis. Clin Immunol. (2001) 98:378–82. doi: 10.1006/clim.2000.4983 11237562

[191] Hull CM Liddle M Hansen N Meyer LJ Schmidt L Taylor T. Elevation of IgA anti-epidermal transglutaminase antibodies in dermatitis herpetiformis. Br J Dermatol. (2008) 159:120–4. doi: 10.1111/j.1365-2133.2008.08629.x 18503599

[192] Sankari H Hietikko M Kurppa K Kaukinen K Mansikka E Huhtala H. Intestinal TG3- and TG2-specific plasma cell responses in dermatitis herpetiformis patients undergoing a gluten challenge. Nutrients. (2020) 12. doi: 10.3390/nu12020467 PMC7071213 32069794

[193] Rose C Dieterich W Brocker EB Schuppan D Zillikens D. Circulating autoantibodies to tissue transglutaminase differentiate patients with dermatitis herpetiformis from those with linear IgA disease. J Am Acad Dermatol. (1999) 41:957–61. doi: 10.1016/S0190-9622(99)70253-7 10570380

[194] Boch K Heck F Hammers CM Antiga E Caproni M Juhl D. Serum reactivity in dermatitis herpetiformis: an international multicentre study. Clin Exp Dermatol. (2023) 49:53–7. 10.1093/ced/llad319 37793183

[195] Karell K Louka AS Moodie SJ Ascher H Clot F Greco L. HLA types in celiac disease patients not carrying the DQA1*05-DQB1*02 (DQ2) heterodimer: results from the European Genetics Cluster on Celiac Disease. Hum Immunol. (2003) 64:469–77. doi: 10.1016/S0198-8859(03)00027-2 12651074

[196] Erlichster M Bedo J Skafidas E Kwan P Kowalczyk A Goudey B. Improved HLA-based prediction of coeliac disease identifies two novel genetic interactions. Eur J Hum Genet. (2020) 28:1743–52. doi: 10.1038/s41431-020-0700-2 PMC7785002 32733071

[197] Ciacci C Ciclitira P Hadjivassiliou M Kaukinen K Ludvigsson JF McGough N. The gluten-free diet and its current application in coeliac disease and dermatitis herpetiformis. United Eur Gastroenterol J. (2015) 3:121–35. doi: 10.1177/2050640614559263 PMC4406897 25922672

[198] Reunala T Blomqvist K Tarpila S Halme H Kangas K. Gluten-free diet in dermatitis herpetiformis. I. Clinical response of skin lesions in 81 patients. Br J Dermatol. (1977) 97:473–80. doi: 10.1111/j.1365-2133.1977.tb14122.x 588461

[199] Fry L Seah PP Riches DJ Hoffbrand AV. Clearance of skin lesions in dermatitis herpetiformis after gluten withdrawal. Lancet. (1973) 1:288–91. doi: 10.1016/S0140-6736(73)91539-0 4119171

[200] Mansikka E Hervonen K Kaukinen K Ilus T Oksanen P Lindfors K. Gluten challenge induces skin and small bowel relapse in long-term gluten-free diet-treated dermatitis herpetiformis. J Invest Dermatol. (2019) 139:2108–14. doi: 10.1016/j.jid.2019.03.1150 30998982

[201] Haffenden GP Blenkinsopp WK Ring NP Wojnarowska F Fry L. The potassium iodide patch test in the dermatitis herpetiformis in relation to treatment with a gluten-free diet and dapsone. Br J Dermatol. (1980) 103:313–7. doi: 10.1111/j.1365-2133.1980.tb07250.x 7426428

[202] Bieber K Kridin K Emtenani S Boch K Schmidt E Ludwig RJ. Milestones in personalized medicine in pemphigus and pemphigoid. Front Immunol. (2020) 11:591971. doi: 10.3389/fimmu.2020.591971 33505392 PMC7829330

[203] Holtsche MM Boch K Schmidt E. Autoimmune bullous dermatoses. J Dtsch Dermatol Ges. (2023) 21:405–12. doi: 10.1111/ddg.15046 37070500

